# Observation of phase correlations in ferrofluids

**DOI:** 10.1038/s41598-026-45834-1

**Published:** 2026-04-05

**Authors:** Alessandro Chiolerio, Giuseppe Vitiello, Mohammad Mahdi Dehshibi, Marco Crepaldi, Diego Torazza, Andrew Adamatzky

**Affiliations:** 1 Bioinspired Soft Robotics, Istituto Italiano di Tecnologia, Via Morego 30, 16163 Genova, Italy; 2 Unconventional Computing Laboratory, University of the West of England, Coldharbour Lane, BS16 1QY Bristol, England, UK; 3 Dipartimento di Fisica “E.R. Caianiello”, Universitá di Salerno, Via Giovanni Paolo II 132, 84084 Fisciano, Italy; 4 Electronic Design Laboratory, Istituto Italiano di Tecnologia, Via Melen 83, 16152 Genova, Italy; 5 Mechanical Workshop, Istituto Italiano di Tecnologia, Via S. Quirico 19d, 16163 Genova, Italy

**Keywords:** Ferrofluid, Colloidal entanglement, Coherent states, Microwave impedance spectroscopy, Learning, Nanoscience and technology, Physics

## Abstract

Magnetic fluids, also known as *ferrofluids*, are excellent candidates for several important research fields, including soft robotics and biomedicine. In this study, we report on the observation of phase correlations between two physically isolated and electromagnetically shielded volumes of ferrofluid. A “twinning” pre-conditioning process, involving the application of hysteresis cycles to the entire fluid volume, was performed to induce a coherent state. Subsequently, the fluid was divided into two separate, shielded containers, with one subjected to an electrical stimulus. We observed statistically significant correlations in the impedance fluctuations between the stimulated and non-stimulated samples, even at a separation distance of up to 10 meters. These correlations persist for approximately 100 hours under laboratory conditions and were consistently observed in both water-based and hydrocarbon-based ferrofluids within a temperature range of 10–50 °C. The experimental design excludes classical electromagnetic fields as the mediating force. These findings suggest the presence of a long-range, collective phenomenon in ferrofluids, opening new avenues for investigating complex interactions in colloidal systems.

## Introduction

Ferrofluids (FFs) are composed of nanometric-size superparamagnetic particles suspended in a solvent and exhibit a range of collective behaviours due to the unique properties of these particles^1,2^. The most remarkable collective behavior of the FFs consists of a strong response to the magnetic field triggered, for example, by a permanent magnet, which produces a spiky, dynamically responsive set of cones emerging from the flat surface of the liquid. Their superparamagnetic nature, often derived from magnetite for its responsiveness, allows FFs to be used in a diverse array of applications, including rotary seals^3^, dampers^4^, bearings^5^, lubricants^6^, heat transfer media^7^, soft robotics^8,9^ and neuromodulation of the brain^10^. Central to the dynamic interactions within FFs is the magnetic dipolar interaction, which scales inversely with the cube of the distance ($d^{-3}$) between particles, facilitating a strong coupling across the multitude of dispersoids. Our exploration extends beyond the potential applications of FFs to explore some uncharted collective effects. Previous investigations verified the in-memory computing capabilities of FFs^11^, the learning pathways in liquids^12^, Pavlovian conditioning^13^, and ultimately led to the identification of a repeatable conditioning effect between physically separate FF samples. Intriguingly, this effect persists even when possible interactions between the samples and electromagnetic reciprocal influences are meticulously excluded, hinting at an underlying phase correlation (collective) mechanism. The observed conditioning effect we are investigating in this paper aligns indeed with the concept of entanglement, the correlation between spatially disjoint objects^14–16^. Entanglement has challenged the traditional notions of locality and reality, among the foundations of observed objectivity^17,18^. Our findings suggest that the entanglement between two *twin* samples of FF, involving a vast amount of particles (we typically work with $2 \times 10^{18} L^{-1}$ particles, corresponding to a volume fraction of 0.2602), can induce phase correlations among them, thus presenting a colloidal entanglement. It is known that magnetic nanoparticles in FFs experience long-range dipole-dipole interactions, significantly influencing their collective properties. For instance, the interaction strength depends on the particles’ magnetic moment and spatial arrangement within the fluid. Interactions lead to aggregation and chain-like formations, altering the material’s rheological and magnetic behaviors. As discussed in detail in^19^, some water-based highly concentrated FFs, such as those here used, feature a robust interaction and consequently have a much higher blocking temperature in comparison to other commercial FFs, a relaxation that does not show the typical Brownian peak at smaller frequency (in the 1 to 100 kHz range) and interparticle distances, that can be smaller than the particle radius (which is our case). Therefore, important conditions are met for experiencing strong interparticle interaction. Furthermore, colloids exhibit phase behaviour analogous to atoms in a crystal^20^. This colloidal perspective is particularly interesting since it extends the observation of quantum entanglement to many-particle systems^21–24^. Our results are achieved using cost-effective equipment and materials operating under standard laboratory conditions. The large number of elements per unit volume creates a significantly expanded Hilbert space associated with their degrees of freedom, offering exciting possibilities in information processing and beyond^25^. To evaluate the diversity in statistics of samples, we adopt the Kullback-Leibler divergence^26^. Adapting these methods to FFs through matrix calculus allows for a nuanced exploration of the entanglement phenomena we observe, involving Machine Learning (ML)^27–29^ approaches that become necessary when the amount of collected data is too large for manned interpretation. We differentiate between intra- and inter-system entanglement. Defining the system’s boundary is critical in setting the boundary conditions for our experiments. When the boundary is defined as the outer surface of a specific volume of fluid, say 1 mL, anything related to the particles floating within the vial is considered intra-system. When conditioning 2 mL of fluid with a particular procedure, we have called “twinning”, separating it into two halves and placing it into two identical containers, anything that relates the particles between the two vials is considered inter-system. This “twinning” is nothing but a common electromagnetic history received by the FF to (pre)condition its dynamical state, creating a “standard” procedure that can be repeated and verified under various experimental conditions, such as temperature, distance, signal amplitude, and stimuli sequence, among others. Worth noting that the impedance of an FF is certainly affected by temperature^30^, magnetic field^19^, and also electric field^31^. The “twinning” procedure establishes slow electrical fluctuations that create the conditions for setting a coherent state between the dispersed magnetic nanoparticles, characterized by spontaneous oscillations^32^, until relaxation occurs. Such a coherent state is then exploited to demonstrate phase collective features. Also note that the preconditioning by itself, performed separately on each of the two vials, does not suffice to create the conditions for phase correlation.

Colloids, having characteristic lengths on the order of hundreds to thousands of nm, exhibit Reynolds numbers that are significantly lower than 1, meaning that inertial forces are negligible compared to viscous forces. The characteristic length is the length scale that sets the flow around the object of interest; for colloids, this is usually the particle hydrodynamic radius^33^, which is in the specified range. As described by the scallop theorem^34^, no momentum can be accumulated moving in such a fluid. Therefore, the dissipation of any structure resulting from the ordering, eventually induced by conditioning fields, is not instantaneous, emphasizing the memory of the colloidal arrangements (see Supplementary Information, Sect. 1). We have found several colloidal systems featuring a memory, namely a suspension of polyaniline nanorods^35^, graphitic carbon nitride^36^, zinc oxide nanoparticles^12^ and ferrofluids: a water-based system containing magnetite nanoparticles can provide a slowly fading memory, that can also be profitably used to implement computational schemes, such as reservoir computing^11^.

In many-body physics, the competition “between exchange interactions and disorder”^37^ may lead to ordering when the spontaneous breakdown of symmetry occurs, with the formation of in-phase long range correlations among the system’s components (i.e., the magnetic moments of FF nanoparticles, in our case), as stated by the experimentally widely confirmed Goldstone theorem^38–41^. An equivalent many-body configuration was proven to induce quantum effects at large scale in 1985 work by Devoret, Martinis and Clarke^42^, leading 40 years later to the Nobel prize assignment. Memory effects in highly correlated many-body systems arise when ergodic mixing is hindered, so that information about initial conditions persists in observables over times much longer than expected. Spatially, these memories are typically quasi-local and domain-based^43,44^. Our choice of using the electrical field instead of the magnetic one, in the (pre-)conditioning of the samples, is due to the presence of a huge number of electrical dipoles in the ferrofluid. To explain how an electric field can create a correlation of magnetic moments, consider that, as said above, it triggers long-range correlations among the electrical dipoles in the FF, with variation in time of the electric polarization. This is a source of the curl of the magnetic field $\textbf{B}$ (cf. the corresponding Maxwell equation^45^), which produces the breakdown of the rotational symmetry of the nanoparticles’ magnetic moments, their consequent long-range correlation, and magnetic polarization.

One remarkable feature of our finding is the correlation, or entanglement, within a complex and dense collection of dipoles^46^. The phenomenon of entanglement dominates our analysis and results, and much of our discussion is devoted to it in the following sections. Another distinctive aspect of our study is the manifestation of fractal properties of the FF dynamics (cf. especially the discussion in Sect. Comparing **UN** and **TW** datasets). The interest in fractals lies in the fact that an isomorphism exists between fractal self-similarity and coherent states^41,47,48^. Detecting fractal self-similarity thus signals that coherent states occur in the ferrofluid. The fractal dimension, which, as well known, is given by the slope of the linear fitting in the log-log plot of the physical quantities to which coherent states are related, provides a measure of the degree (q-deformation, technically) of coherence.

Notice that, in the expression *phase correlation*, the *phase* is the one of the complex valued field denoting the ‘correlated’ elementary components, e.g., in superconductivity, the electron field is given by $\psi = \exp ^{i \theta } \phi$^39,40,49^, where the ‘phase’ $\theta$ is the Goldstone quantum field, associated (through the De Broglie relation) to the correlation between electrons in the superconductive state. A different meaning is the one in the expression *phase transition*, where the ‘phase’ denotes the ‘dynamical regime’, and the ‘transition’ is through different dynamical regimes, for example, from the ferromagnetic dynamical regime to the non-ferromagnetic one. The proper meaning in which the word ‘phase’ is used is clarified by the context.

In the following sections, we describe the equipment, software and data collection tools (Sect. “Methods”). We present how the experiments are performed via voltage stimuli and impedance readings (Sect. “How the experiment is performed: the voltage stimulation and the Microwave Impedance Spectroscopy readout”), define the physical parameters space we have explored (Sect. “The physical parameter space”), and describe the logic process to validate the findings (Sect. “From null hypothesis, to false hypothesis and statistically relevant dependencies”). We discuss the findings based on the complex impedance state measures of FF volumes and the fractal self-similarity (Sect. “Comparing **UN** and **TW** datasets”), and we provide further elements of discussion (Sect. “Further elements”). Conclusions are presented (Sect. “Conclusions”).

## Methods

### Conditions

All measurements are performed in an electronic laboratory environment at room temperature (unless otherwise specified), and they are executed without people around to avoid possible vibrations or interferences that may occur in the laboratory during normal working hours.

### Ferrofluids

We have used an EMG601P ferrofluid, FerroTec, Lot Number U021920A^50^. The properties revealed by the manufacturer are: saturation magnetization 44*mT* (the highest among research-grade products), viscosity lower than 5 mPas at a temperature of $25 ~\deg C$, density $1.34 ~gmL^{-1}$, pH between 8.5 and 9, cationic surfactants of proprietary formulation between 6 and 22 volume %, magnetite nanoparticles between 1.2 and 7.9 volume % (that corresponds to a maximum mass fraction of 31.6%). As reported in the Materials Safety Data Sheet, “the precise composition of this mixture is proprietary information”. For a complete chemo-physical analysis of this FF, we refer to^51^, including SEM, TEM, HRTEM, XRD, SAED, XPS, ATR-FITR. TEM images of dried particles revealed their diameter distribution: the smallest particles detected were 6 nm (<5% detection frequency), the biggest 32 nm (<2% detection frequency), the most abundant 14 nm (<18% detection frequency). Approximately 95% of the detected particles are in the range from 6 to 24 nm. The quantity of liquid used is 4 *mL*, released in the vial using a pipette (unless otherwise indicated). The hydrocarbon-based ferrofluid we have used to test the effects of other solvents is the EFH3, FerroTec, Lot Number R012517A. The properties revealed by the manufacturer are: saturation magnetization 65 *mT*, viscosity 12 *mPas*, density $1.21 - 1.42 ~gmL^{-1}$, oil soluble dispersant between 6 and 30 volume %, magnetite nanoparticles between 3 and 15 volume %. As reported in the Materials Safety Data Sheet, “Contains: DISTILLATES (PETROLEUM), HYDROTREATED LIGHT” and “The precise composition of this mixture is proprietary information”.

### Reservoir

**Fig. 1:**
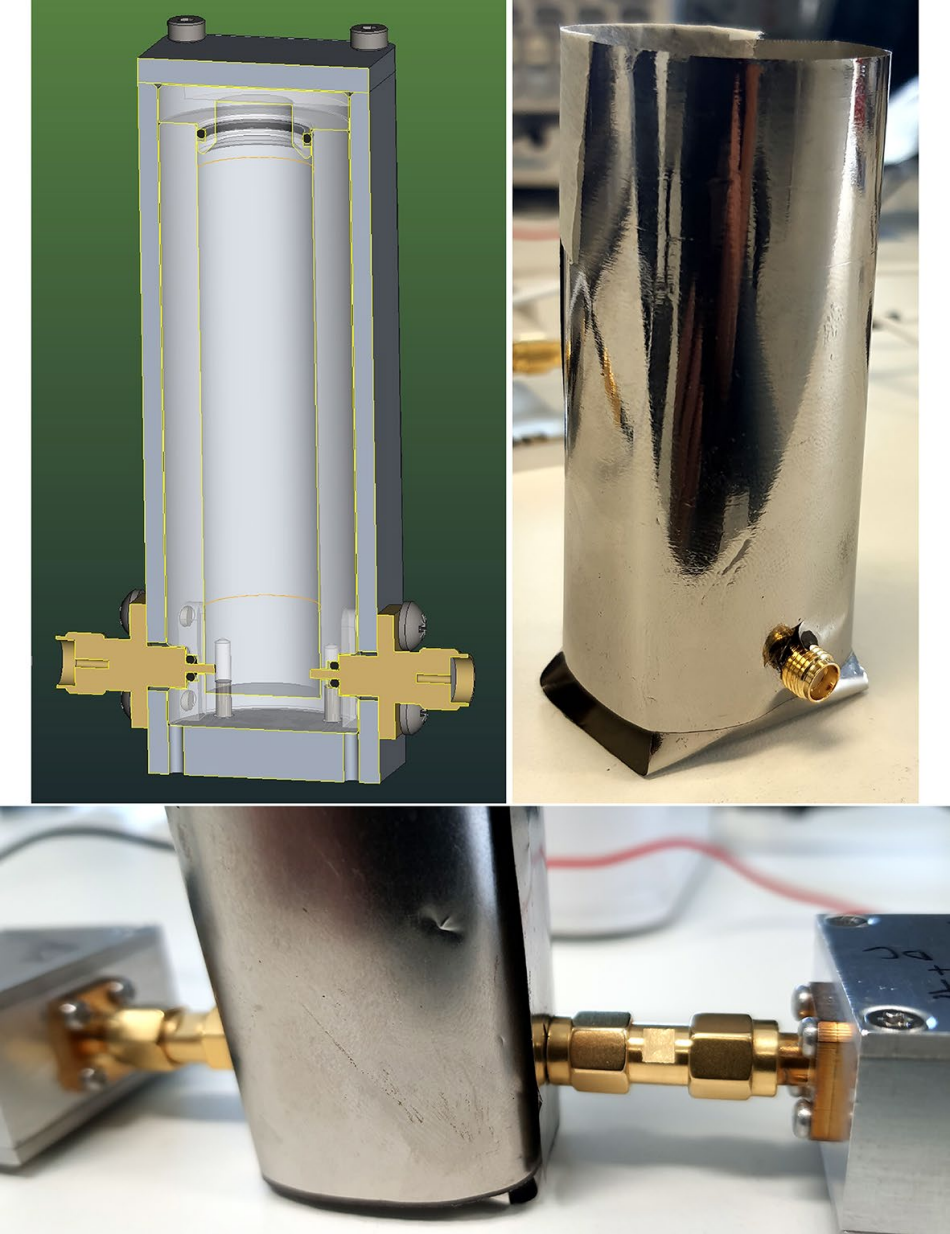
Bisected view of the FF reservoir (top left). Picture of the permalloy coating assembly during the preparation of the reservoir for measurements (top right). Picture of reservoir A during measurements, showing SMA bridges connecting the bias tees (bottom).

Two vials are designed to contain the FF and characterize its states. They feature a fixed volume of about 6 cc (maximum). Both the vials are Computer Numerical Control (CNC) machined- using Polytetrafluoroethylene (PTFE)- a teflon material for containing the FF and using AA-7075 Aluminium alloy as the outer shield. Teflon is used as a liner to contain the FF due to its excellent chemical inertness and temperature resistance properties. Moreover, its non-sticky and low-frictional properties are very suitable for handling the FF. AA-7075 is chosen for designing the outer shielding due to its higher strength, low density, better corrosion resistance, and easy CNC machining. Two electrodes of the SMA (SubMiniature version A Wurth Elektronik, 1.6 mm straight PCB, Manufacturer number 60314202124525) connector are submerged inside the FF by which the electrical stimuli are applied. The ground pins of the connectors have been cut to expose only the feed line of the connector. There are seal rings (O-rings) on the electrodes and on the upper lid of the inner cylinder, and this makes the FF completely sealed from the outer environment. The seal rings are made of Nitrile Butadiene Rubber (NBR) polymer. The vial has a total height of 80.5 mm, and the area of the square-shaped base is about 250 mm^2^. The bisected and the real view of the vial are shown in Fig. 1. The diameter of the inner cylinder is 10.8 mm, and its height is 54 mm, excluding the inverted cone-shaped bottom part. The part of the electrodes of the SMA connectors submerged into FF is about 0.9 mm. Outside the liner, we have fixed a permalloy metallic glass foil that provides a further shield against magnetic fields, which we might expect to be prominently environmentally friendly.

**Fig. 2:**
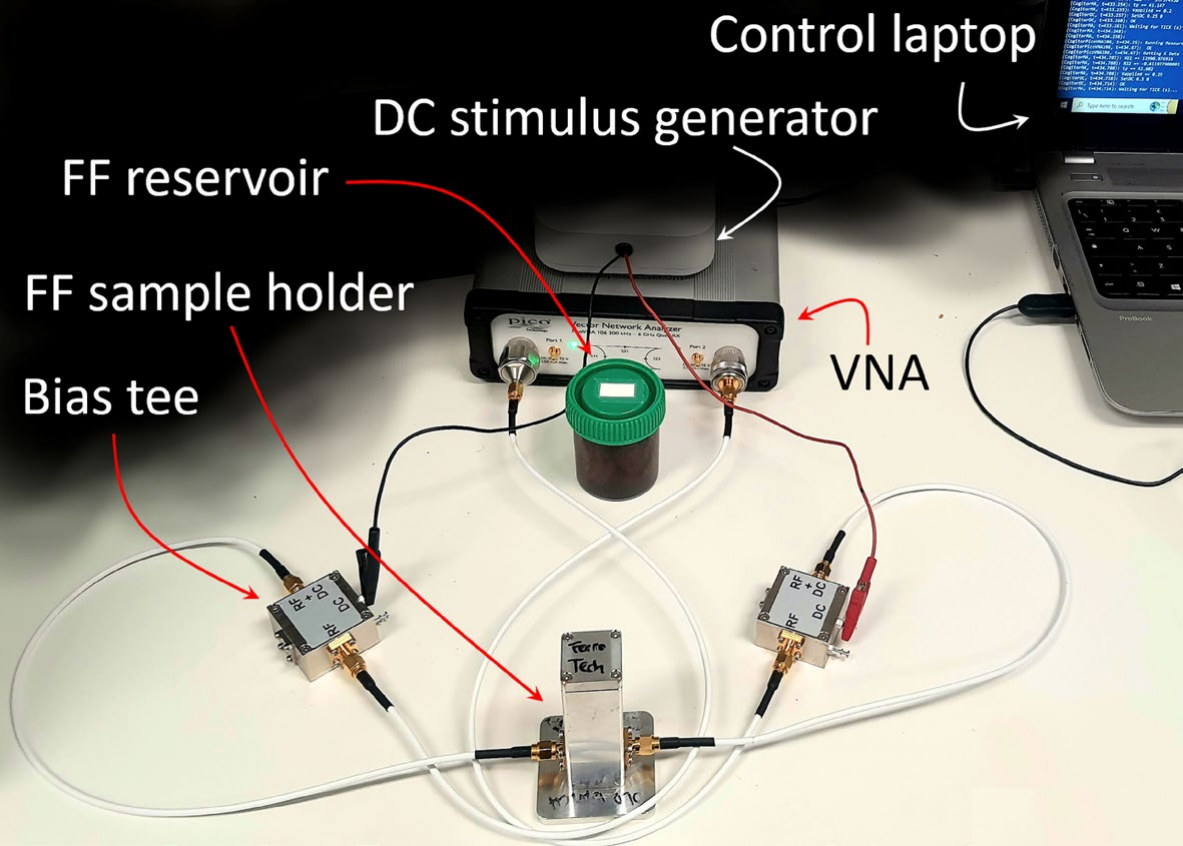
Experimental setup shown during a pre-conditioning phase.

A labeled picture of the complete setup is shown in the next Fig. 2, the photo was shot during one of the pre-conditioning steps, when the FF drawn from the original reservoir is put into a sample holder and submitted to cyclic voltage sweeps, before being split into two sample holders.

### Vector network analyzer

A PicoVNA 106 (300 kHz–6 GHz), Pico Technology, UK, has been used to read out the status of the material, using its built-in Dynamic Link Libraries (DLL) under Microsoft Windows 7. In our experiments, the RF power used to perform the frequency sweep by the VNA is -3 dBm, and we have not observed any significant impact of such a signal on the internal status evolution of the liquid. The number of measurement points was 201.

### Bias Tee

The two bias tees used are commercial TCBT-14+, Mini Circuits (10 MHz–10 GHz), that have been soldered on two custom PCBs designed to be mounted on the RF mini enclosure RF-ENCL-MINI-NF-01, Gequipment.

### DC/AC generation

We have implemented the DC generator using a Micropython Board V1.1 with an internal charge pump (MAX 44267 EVAL KIT). The AC arbitrary function generator is a Tektronix AFG 31052.

### Measurement software

The measurement software runs on a Windows 7 Virtual Machine, installed on a CentOS 7 control domain. Both VNA and DC generators are connected to the PC using USB cables.

To reproduce the measurements in the manuscript, it is sufficient to write a program that coordinates both the VNA and DC generator to read out the S-parameters from the liquid and set the DC bias point. In this work, however, we have designed a specific Python scripting language that executes and compiles specific experiment files.

### Impedance parameters calculation

To calculate the impedance parameters starting from the S-parameters, we have used the following Eqs. (1–4),

1 $$\begin{aligned} Z_{11}&= \frac{(1+S_{11})(1-S_{22})+S_{21}S_{12}}{\Delta _S}Z_0,\end{aligned}$$

2 $$\begin{aligned} Z_{12}&= \frac{2S_{12}}{\Delta _S}Z_0,\end{aligned}$$

3 $$\begin{aligned} Z_{21}&= \frac{2S_{21}}{\Delta _S}Z_0,\end{aligned}$$

4 $$\begin{aligned} Z_{22}&= \frac{(1-S_{11})(1+S_{22})+S_{21}S_{12}}{\Delta _S}Z_0, \end{aligned}$$

where $\Delta _S = (1-S_{11})(1-S_{22})-S_{21}S_{12}$. The above equations’ output is the impedance complex numbers values over frequency, from which magnitude values can be extracted. In our measurement system, these calculations are computed during the S-parameter measurements, but can be calculated offline. We have assumed $Z_0$ = 50 $\Omega$. In our tests, we have not eliminated the contribution of the vial, but we have considered its full impedance contribution, including the colloid. Next we compute the Riemann integral average (Eq. 5) which represents an optimal choice; here $Z_{ij}(\omega )$ is a complex-valued impedance, function of the angular frequency $\omega$ in the range comprised between $\omega _{Max}$ and $\omega _{min}$:

5 $$\begin{aligned} Z_{ij}^{M} = \frac{1}{\omega _{Max}-\omega _{min}} \int ^{\omega _{Max}}_{\omega _{min}} Z_{ij}(\omega ) d \omega \end{aligned}$$

The Riemann integral (average) features additivity and linearity. Equation 6 defines the engineering parameter $Z_{ij}^{C}$.

6 $$\begin{aligned} Z_{ij}^{C} = \int ^{\omega _{Max}}_{\omega _{min}} |Z(\omega )_{ij} |d \omega \end{aligned}$$

### Controlled temperature tests

The temperature tests were run using a Binder MK53 climatic chamber, staging both vials, RF cables, and a bias tee inside. The remainder of the setup was kept outside the chamber. Once the temperature is set, the system pumps air inside the chamber to provoke thermalization of objects contained in it. We allowed 4 hours as settling time for optimizing thermalization before starting any measurements. Typical experiments are run at 10 and 50 °C. See further discussion on temperature in Section 5, “Temperature effects” of the Supplementary Information file.

### Statistical analysis

The impedance measurements reported here are acquired by collecting massive data for over 90 minutes for each experiment. The reduced space of three S parameters (11, 12, and 22 components) has been used to extrapolate linear fits from bilogarithmic graphs. All data has been processed without excluding any outlier, using a $1^{st}$ order Savitzki-Golay smoothing function over 51 points before generating bilogarithmic plots. Each experiment generates 4.000 points for every component of the scattering matrix (therefore 12.000 points in total), measured over 1.5 h. The sample size is indicated in each caption. The significance of each experiment has been evaluated using Partial Least Squares (PLS) and Principal Component Analysis (PCA), utilising OriginLab® 9.1 (https://www.originlab.com/) and Matlab® R2021b software (https://matlab.mathworks.com/).

## How the experiment is performed: the voltage stimulation and the Microwave Impedance Spectroscopy readout

**Fig. 3:**
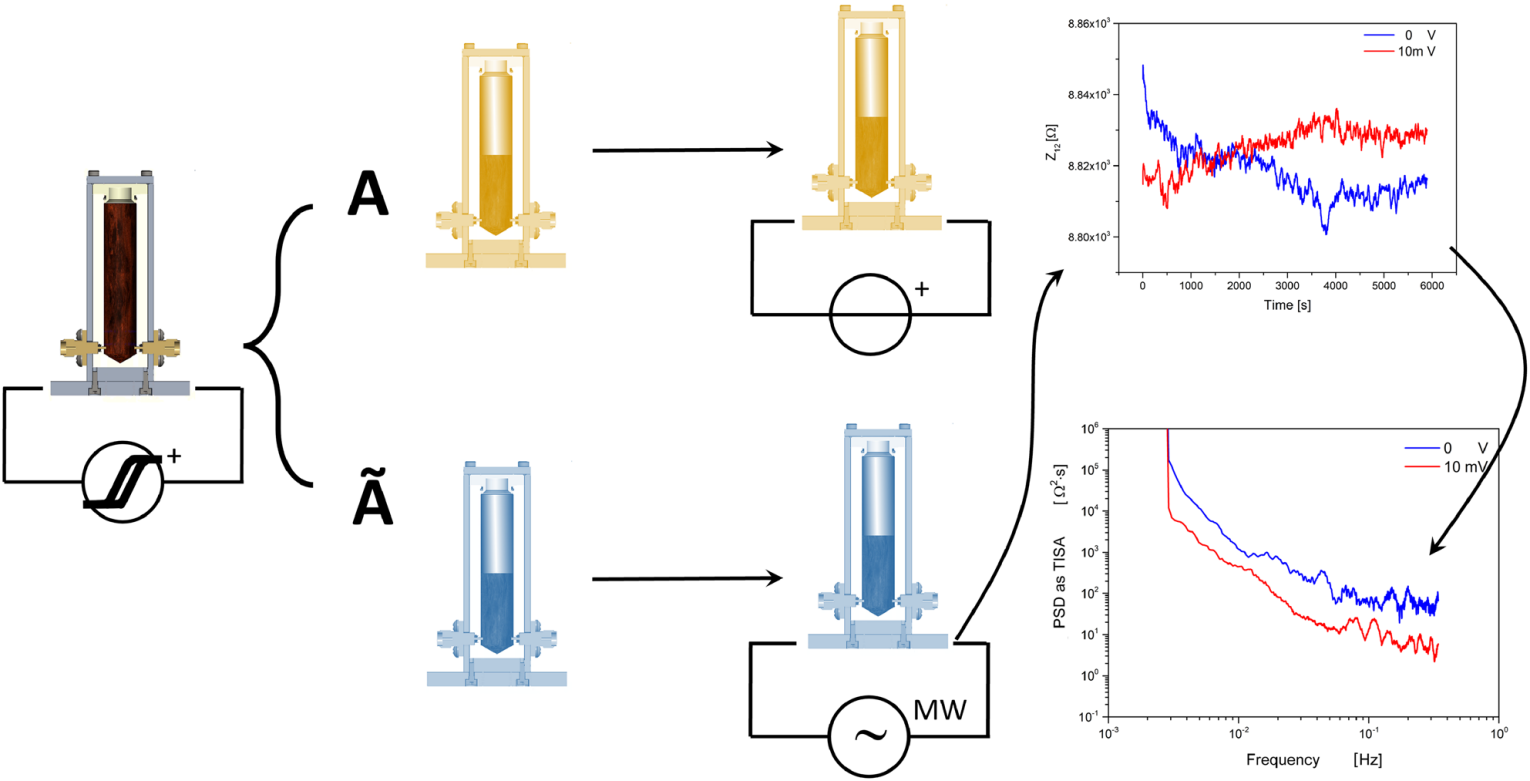
Sketch of the experiment: the entire FF volume undergoes a pre-conditioning operation, consisting in the repetition of a hysteresis cycle for 25 times on a single vial filled with a volume of 4 mL, the “twinned” state (“untwinned” when this is skipped). Subsequently, the FF is split into two halves: A, which undergoes a voltage stimulus, and $\tilde{A}$, which undergoes impedance microwave spectroscopy. Please note that the transparent vials are not representative of real devices (see Supplementary Information S1). The evolution of $Z_{1,2}^{C}$ transmission component of the scattering matrix is shown for two different voltage stimuli (blue: 0 V, red: 10 mV, 4.000 points sample size entirely shown), as well as the fluctuation spectrum comparison, in a bi-logarithmic scale. The Power Spectral Density (PSD) of raw data fluctuations is plotted as TISA (Time-Integral Squared Amplitude), corresponding to the integral under the curve defined by the square of the raw data against time, measured in $\Omega ^{2} s$. The entire sample of 4.000 points is shown for each curve.

The experimental setup we have adopted is simplified in the scheme shown in Fig. 3. In a first phase, a sample reservoir (“A”) is filled with 4 mL of FF.

The total volume of FF is then submitted to 25 voltage cycles, during which the stimulus applied is continuously cycled between -1 and +1 V with a step of 10*mV*. This pre-conditioning operation is quite slow and is completed in 3.5 h. It is worth mentioning that we have verified experimentally that shorter cycling (less than 25 cycles) in the pre-conditioning process did not successfully create the twinning conditions, on the one hand, and that much longer cycling (more than 25 cycles) was not any better in twinning strength. The particular choice of voltage, leading to an electric field of 1 $\hbox {Vcm}^{-1}$, is negligible when compared to electric fields whose effects have been observed inducing structural changes in FFs (in the order of some $\hbox {kVcm}^{-1}$^52^). Nevertheless, as it has been observed, the electric field may act on the nanoparticle ordering close to the electrodes^31^. The reservoir “A” is then opened and half of its volume (i.e. 2*mL*) is poured onto an empty and clean reservoir, marked “$\tilde{A}$”. The two reservoirs are now treated independently, as one is submitted to the voltage stimulus (“A”), while the other (“$\tilde{A}$”) is submitted to wide-band impedance measurements continuously. Microwave Impedance Spectroscopy data consists of the four complex-valued components of the scattering matrix, which are then stored. Scattering parameters $S_{ij}$ relate the backward voltages measured at the two ports of each vial with the forward voltages injected and represent the electromagnetic signal transported across the sample when $i\ne j$ and the electromagnetic signal reflected by the sample when $i=j$. Therefore $Z_{12},Z_{21}$ are said transport components and $Z_{11},Z_{22}$ are said reflection components. Furthermore, $\forall i \ne j, Z_{ij}=Z_{ji}$, in other words, the FF behaves as a reciprocal device: the transmitted signal travels across the liquid regardless of the propagation direction. Data are compressed by calculating the Riemann integral over the entire frequency range for each of the scattering components, instead of maintaining a resolution in frequency and therefore reducing complexity for both visualization and further processing. As we are particularly interested in the information conveyed by fluctuations, we compute the Power Spectral Density (PSD) as Time-Integral Squared Amplitude (TISA) and plot it on a bi-logarithmic scale.

Taking advantage of the linear trend found in the bi-logarithmic plots of TISA versus frequency, particularly in the lower frequency range, we performed linear fits, extracting slope *m* and intercept *q* of the fitting curves, their associated errors $std_m$ and $std_q$, and the relative mean for each parameter:

7 $$\begin{aligned} & \alpha _{m} = \frac{m-\langle m \rangle }{std_m}, \end{aligned}$$

8 $$\begin{aligned} & \alpha _{q} = \frac{q-\langle q \rangle }{std_q}, \end{aligned}$$

This set of quantities is then associated with the physical parameters of each experiment and further processed using multivariate statistical analyses, using two well-known frameworks: the Partial Least Squares (PLS) method and the Principal Component Analysis (PCA). PLS is used to perform prediction and data reduction and requires having both independent and dependent variables from a dataset. Variable Influence on Projections (VIP) is used to determine each predictor variable using the mean-variance in responses, providing, most importantly, a confidence level below which the predictor variable is not significant. Similarly, PCA is used for data reduction and reveals unsuspected dependencies in large datasets. Extracted principal components are linear combinations of variables that provide the highest variance.

## The physical parameter space

**Fig. 4:**
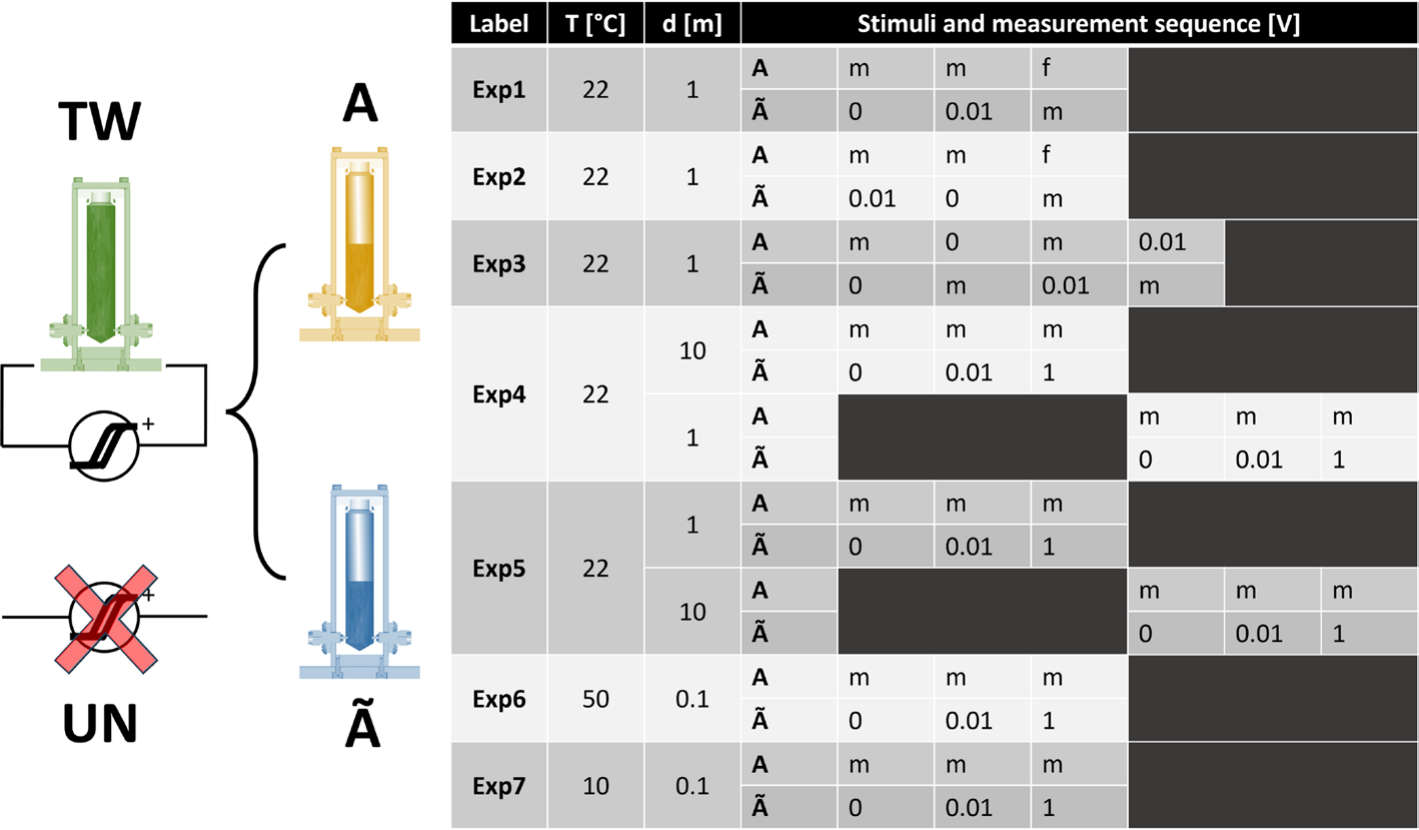
Left: simplified sketch of the “twinned **TW**” (“untwinned **UN**”) states, where the full volume of ferrofluid is (not) submitted to repeated hysteresis cycles and then equiparted in two reservoirs. Right: schematic table of all the experiments performed for both the TW and UN states. Column “T” indicates the temperature, column “d” the distance between vials, and in the “Stimuli and measurements sequence” portion of the table we adopted the following codes: a number indicates the DC voltage stimulus, “m” indicates the impedance measurement, “f” indicates a floating condition, where the vial is put at rest without any stimulus applied.

The experiments performed cover a broad range of situations, including the following parameters: amplitude of the voltage stimulus, specific sequence of stimuli, temperature, distance between reservoirs, and proportions of liquid in the two vials. Each of the experiments was repeated by performing a twinning phase (**TW**) before the characterization, and repeated without performing the twinning (untwinned case, **UN**), cleaning the sample holders, and using fresh FF from the reservoir batch after every passage. A schematic overview of the experiments is summarized in the following Fig. 4, where the particular sequence of stimuli and impedance measurements acted on the vials is shown, together with the other physical parameters.

The rationale behind the choice of the temperature range reflects the limits given by the physical properties of our functional liquid, where most of the content is water. Therefore, we cannot approach temperatures lower than the freezing point of water (hence the choice of 10 °C as the lowest temperature). Similarly, we cannot approach temperatures close to the boiling point of water. Furthermore, the generation of steam could represent a harmful situation, since the reservoirs are sealed and do not allow volume changes; therefore, for safety reasons, we chose 50 °C as the highest temperature. The twinning phase is always performed at the same conditions as the measurements, 22 °C and 101 kPa. The overall time required to perform all the experiments and collect measured data has been approximately 120 h.

## From null hypothesis, to false hypothesis and statistically relevant dependencies

We now introduce the logic structure of the experimental activity performed to establish or refute the actual existence of the phase correlation observed in FF.

- Null hypothesis 1): the voltage-induced phase correlation does not exist. This implies that there is no statistically relevant effect between separated vials, regardless of their preconditioning state. This hypothesis and related experiments are discussed in Sect. “Comparing **UN** and **TW** datasets”, where we compare the two datasets (**UN** and **TW**) collected in all of the experimental conditions (parametrising distance, voltage, temperature) and conclude that the voltage-induced phase correlation taking place in the **TW** dataset does exist.
- False hypothesis 2): if the voltage-induced phase correlation between separated vials in the **TW** state exists, then the null-voltage-induced effect between separated vials in the **TW** state does not exist. Null-voltage means that the vial connected to the DC stimulus is not submitted to any voltage. This experiment is discussed in Sect. “Further elements”.
- Hypothesis 3): the voltage-induced phase correlation observed in the **TW** dataset depends only on the twinning procedure. This hypothesis is discussed in Sect. “Further elements”, where we perform the preconditioning separately on both vials and then verify the presence of statistically relevant correlations, concluding that preconditioning separate vials does not enable phase correlations.
- Hypothesis 4): the voltage-induced phase correlation depends on the distance between vials. This hypothesis is discussed in Sect. “Comparing **UN** and **TW** datasets” and in the Supplementary Information file, Section 4. We conclude that there is no statistically significant dependence on the distance for the **TW** dataset, and that there is a statistically relevant dependence for the **UN** dataset, but the amplitude is negligible.
- Hypothesis 5): the voltage-induced phase correlation depends on the temperature. This hypothesis is discussed in Sect. “Comparing **UN** and **TW** datasets” and in the Supplementary Information file, Section 5. We conclude that temperature affects all datasets, however it is not detrimental to the phase correlation whenever they are present.
- Hypothesis 6): the voltage-induced phase correlation depends on the time. This hypothesis is discussed in Sect. “Comparing **UN** and **TW** datasets” and in the Supplementary Information file, Section 6. We conclude that time (ageing) affects all datasets, however it is not detrimental to the phase correlation whenever they are present.
- Hypothesis 7): the voltage-induced phase correlation depends on the FF volume. This hypothesis is discussed in the Supplementary Information file, Section 7. We conclude that the volume affects the measurements: when the stimulated volume is twice the sensed volume, the voltage-induced phase correlation can be observed, while when the stimulated volume is half of it, no effect is observed.
- Hypothesis 8): the voltage-induced phase correlation depends on the nature of the solvent. This hypothesis is discussed in the Supplementary Information file, Section 8. We conclude that the hydrocarbon-based FF shows a phase correlation, operating at lower fluctuation frequencies.

Further elements we bring to the Reader’s attention to highlight the nature of the FF system are included in Sect. “Further elements”, namely: cumulative statistics properties, conjunct probability rules, de Broglie wavelength estimates, and interference tests.

## Comparing **UN** and **TW** datasets

### The fractal dimension of impedance fluctuations

As remarked in Sect. “Introduction”, an isomorphism exists between fractal self-similarity and coherent states. We are thus interested in detecting fractal structures in our measurements since this might signal the presence of coherent dynamical structures in the FF. Our analysis in this section, aimed to such a task, shows that this is indeed the case.

**Fig. 5:**
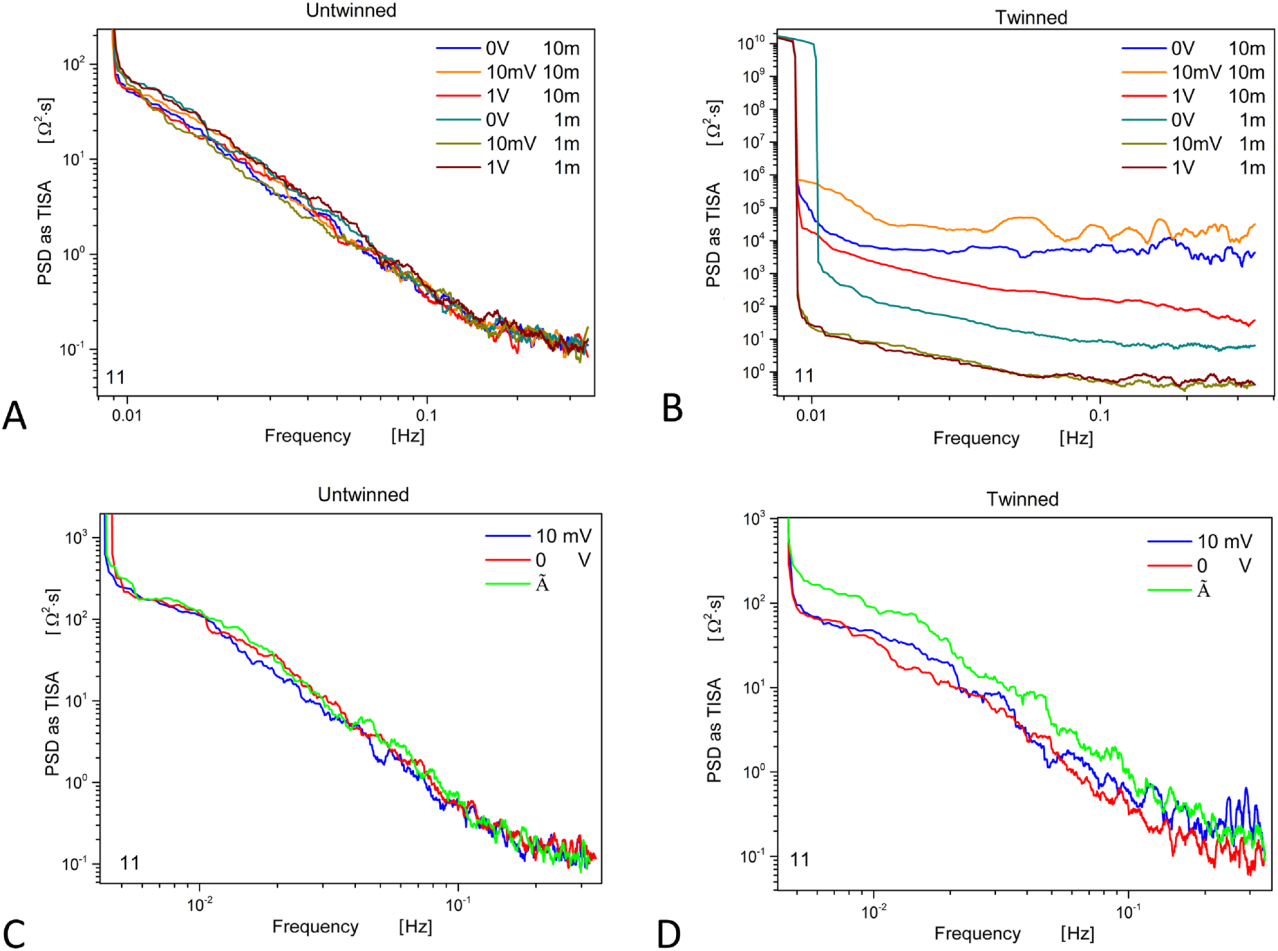
Comparison between scattering matrix reflection parameter 1, 1. Long-range experiment Exp5, untwinned (panel A) and twinned pre-conditioning (panel B). Simple permutation experiment Exp1, untwinned (panel C) and twinned pre-conditioning (panel D). The legend shows the colour code assigned to codify the voltage stimulus amplitude and the distance between vials. The Power Spectral Density (PSD) of raw data fluctuations in the impedance “response” is plotted as TISA (Time-Integral Squared Amplitude) power, corresponding to the integral under the curve defined by the square of the raw data against time. When specified in the legend, the impedance reflection is also measured on sample $\tilde{A}$. The entire sample of 4.000 points is shown for each curve, and an adjacent averaging smoothing of over 51 points was used.

When measuring the scattering matrix temporal fluctuations, the amount of data to be visualized and correlated is too high to allow an easy interpretation (the scattering matrix being composed of four functions). In the following, we will report results about the 1, 1 reflection component of some experiments, to show to what extent the twinning pre-conditioning is fundamental in changing the behavior of the FF reservoirs. Figure 5 shows a comparison between untwinned measurements (left column) and twinned ones (right column) in Exp4 (top row) and Exp1 (bottom row). Some important differences can be immediately spotted.

The power spectral density (PSD) is expressed as Total Impulse Square Amplitude (TISA) and reveals a linear trend in the frequency range explored by our experiments, particularly regular in the low-frequency region. Typically, in the untwinned pre-conditioning state, the fluctuations are quite similar (see panels A and C; the curves collapse in every region of the graph). The low-frequency range, close to $10^{-2}$ Log Hz, is where most likely the stimulus applied to reservoir $\tilde{A}$ produces a measurable “response” effect in the impedance of reservoir A, as an increased PSD. This effect is much more evident when the twinning pre-conditioning is performed (see panel D). The long-range experiment shows remarkable results: all the curves in the untwinned case look similar and overlap in many regions (panel A). In the twinned case (panel B), on the contrary, we observe a strong differentiation in the “response”, particularly when the reservoirs are kept at 10 m apart. Possible perturbations and sources of internal noise may influence the coherence of the original twinning state when situating the reservoirs at different distances. This is signaled by the differences in the plots since the slope of the linear fits and their crossing with the ordinate axis (not shown in the Figure) are related to the coherence deformation^47,48^.

**Fig. 6:**
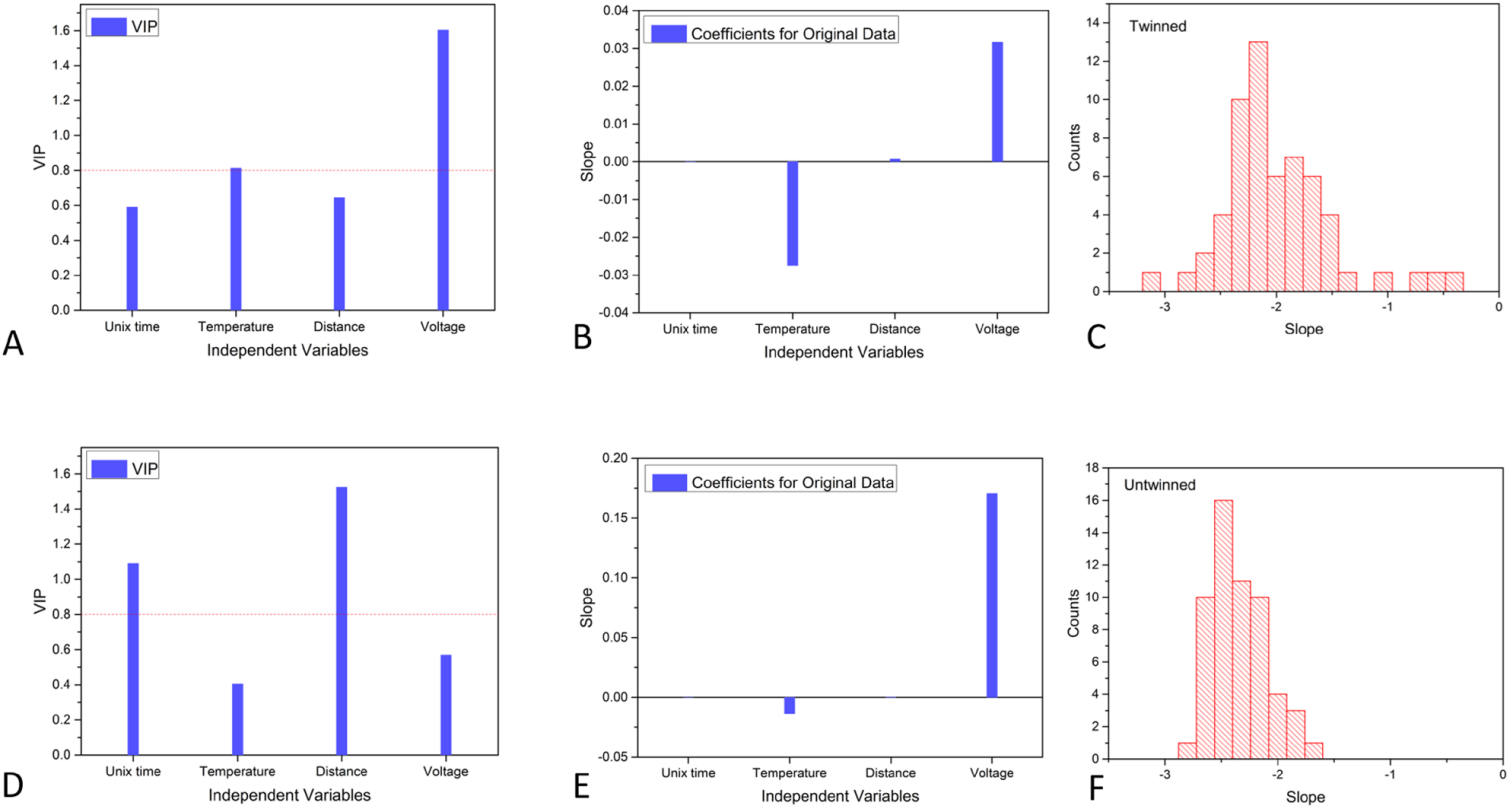
Cumulative statistical analyses of fractal dimensions (linear fit slopes) of all the impedance scattering matrix components. Variable Independence Parameter (VIP) plots for the twinned state (**A**), dependencies of the slope of the linear fit to impedance (**B**), and slope values distribution (**C**). For the untwinned state: VIP plots (**D**), dependencies of the slope (**E**) and slope values distribution (**F**). The statistical sample is composed of 4.32 million entries divided into 60 experiments for each of the two pre-conditioning operations.

Let us now consider the linear fits of such curves in the low-frequency region: we can extract six dependent variables, featuring a certain degree of correlation between themselves: the slope and its standard deviation, the intercept and its standard deviation, and the two associated relative averages (see Eqs. 7 and 8). Said six variables can be calculated for each of the four components of the scattering matrix. At the same time, two of them can be reduced to one as the physical system is almost perfectly reciprocal^11^. The high amount of data (60 measurements for each of the twinned/untwinned pre-conditioning operations, times six variables, times three components, times 4 thousand samples over 1.5 h = 4.32 million real numbers) must be evaluated using PLS and PCA. Figure 6 is extremely important, as it summarises the results of the cumulative statistics on all performed measures and allows to compare directly what happens to the fractal dimension of the impedance fluctuations (i.e. the slopes in Fig. 5) when the pre-conditioning is applied or not: the upper row is related to the twinned case, the bottom row to the untwinned case.

The Variable Independence Plot, also reported as Variable Importance in Projection (VIP) methodology, is a technique to measure and visualize the importance of predictor variables in a supervised Machine Learning (ML) model. It provides insights into how each feature contributes to the predictions, either globally (across the dataset) or locally (for individual predictions). VIP helps to identify which variables most influence the output of a model, enhancing interpretability and guiding feature selection or model refinement. Given a set of predictors $X \in \mathbb {R} ^{N \times K}$ and a set of dependent variables $Y \in \mathbb {R} ^{N \times M}$, the PLS model tries to find a solution in the form $Y=XB+B_{0}$. It is formulated as:

9 $$\begin{aligned} VIP_{k}= \sqrt{\frac{K \cdot \sum _{n=1}^{A} w^{2}_{a,k} SSY_{a}}{A \cdot SSY_{total}}} \end{aligned}$$

where: *K* is the number of independent variables in the predictors *X* (in our case: absolute time, temperature, distance, voltage); *A* is the number of PLS components (in our case: the four components of the scattering matrix); $w_{a,k}$ are the weights of $X_{k}$ in the $a^{th}$ component; $SSY_{a}$ is the sum of squares of the explained variance for the $a^{th}$ component of the dependent variables; $SSY_{total}$ is the total sum of squares explained in all the components. The quantity expressed in Eq. (9) is normalized so values larger than 1.0 indicate important variables. The value of 0.8 is often used as a limit below which the variables are considered unimportant. The VIP plot (shown in Fig. 6 panels A and D) puts in evidence how the stimulus amplitude can be used to predict the dependent variables in each experiment, with a high confidence of 1.6 > 0.8 threshold after the twinning. In other words, we have found the right conditions to influence the fractal dimension of impedance fluctuations of reservoir A by applying a stimulus on reservoir $\tilde{A}$, in a (statistically) predictable manner: such conditions are represented by the pre-conditioning procedure.

On the contrary, when the twinning is not performed, the significance of the relationship between fractal dimension of impedance fluctuations and the independent variables is *relevant* only for the distance between reservoirs and the absolute age of the FF (see again Fig. 6D), but the coefficient is *zero* and therefore there is no dependency. The slope of the linear fit to reflection impedance component is always dependent on the temperature and the stimulus amplitude (see Fig. 6 panels B and E), while only the twinned state is such that said dependency is significant. The statistics of the fractal dimension of impedance fluctuations change slightly in the case of twinning, including values that range between less than −3 to almost 0, while when no twinning is performed, the values are always included in the range −3 to −1.5 (see Fig. 6 panels C and F).

The shape of the distribution has also changed visibly, encompassing a multi-modal distribution when the twinning is performed. When the distribution of slopes is narrower, fractality is limited, and the system under study is more stable. A broader distribution, like the one observed in the case of twinning, points to a higher complexity. It is known that an isomorphism exists between fractal self-similarity and coherent states in QFT^41,47,48^, and therefore, between the fractal dimension and the entropy associated with a coherent condensate. In bilogarithmic plots of conjugate variables (impedance fluctuations Log PSD versus Log f), if fractal self-similarity is present, the measured data follow a distribution along a line, whose slope is related to the fractal dimension. At constant *V* and constant *T*, the system may move along a line of slope $T=dU/dS$ in the plane (*U*, *S*). When coherent condensate densities undergo variations that affect entropy, a change in fractal dimension occurs as well. A zero fractal dimension, observed in some experiments after twinning pre-conditioning, is also a unique feature, corresponding to fluctuations that are stable in the entire frequency range under study (uniform noise).

### Machine-powered analyses of the entire dataset

**Fig. 7:**
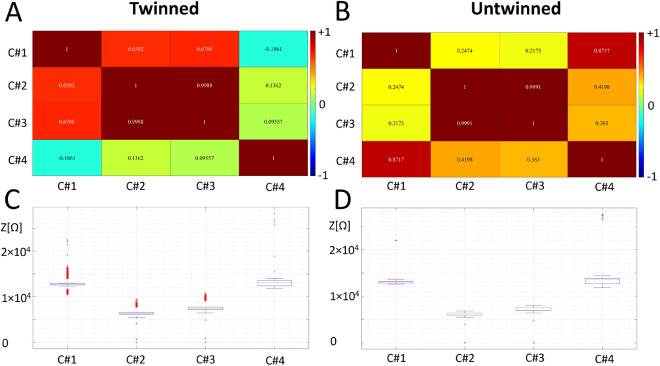
Heatmaps of the twinned (**A**) and untwinned (**B**) pre-conditioning operations, and boxplots of the twinned (**C**) and untwinned pre-conditioning operations (**D**). The “‘components C#n” identified in the axes correspond to the four components of the impedance matrix: C#1 is the reflection component $Z_{(2,2)}$, C#2 is the transmission component $Z_{(2,1)}$, C#3 is the transmission component $Z_{(1,2)}$ and C#4 is the reflection component $Z_{(1,1)}$. The statistical sample comprises 32 million entries divided into 60 experiments for each of the two states. The boxplot is a standard visualization tool that allows to identify Q1 and Q3 (box size), the median (red line in the box), 1.5 times the interquartile range (whiskers above and below the box).

Machine-powered analyses can be performed directly on massive raw data without the need for data compression: in the previous paragraph, we reduced the information to one single scattering matrix component (reflection parameter 1, 1), applied a $1 \mathrm{st}$ order Savitzki-Golay smoothing over 51 points, performed a linear fit in the low-frequency range, and compared the extracted parameters. In the next analyses, instead, we run PCA algorithms comparing two groups of experiments on *all* data, and the results are shown in Fig. 7. Here, we compared cumulatively all the performed experiments belonging to the twinned case to all those belonging to the untwinned case. From the heatmaps, one can appreciate the interdependencies between the four scattering components. Each heatmap relates the four scattering matrix components with the four principal components extracted by ML algorithm, providing the entire range of co-dependencies: +1 (direct proportionality with positive sign), −1 (direct proportionality with negative sign), 0 (no dependency at all), which, beyond the obvious self-correlation of each component with itself, provides an almost perfect match between the two transmission components (reciprocity of the device under test). The first reflection component is highly correlated with the two transport components (above 0.65) and has a small, negative correlation with the second reflection component! The system is, therefore, mostly antisymmetric under reflection. In the untwinned case, there is a lower correlation between any reflection component and any transmission one ($< 0.42$), and most notably, there is a high and positive correlation between the two reflection components! The system is, therefore, mostly symmetric under reflection. The boxplots of the four scattering matrix components at a first glimpse show how similar the measures in the two cases are, while looking with greater attention, one might notice the differences: the medians, the skewness of the distributions (in comparing the median with the box extremes, Q1 and Q3), and the outliers.

**Fig. 8:**
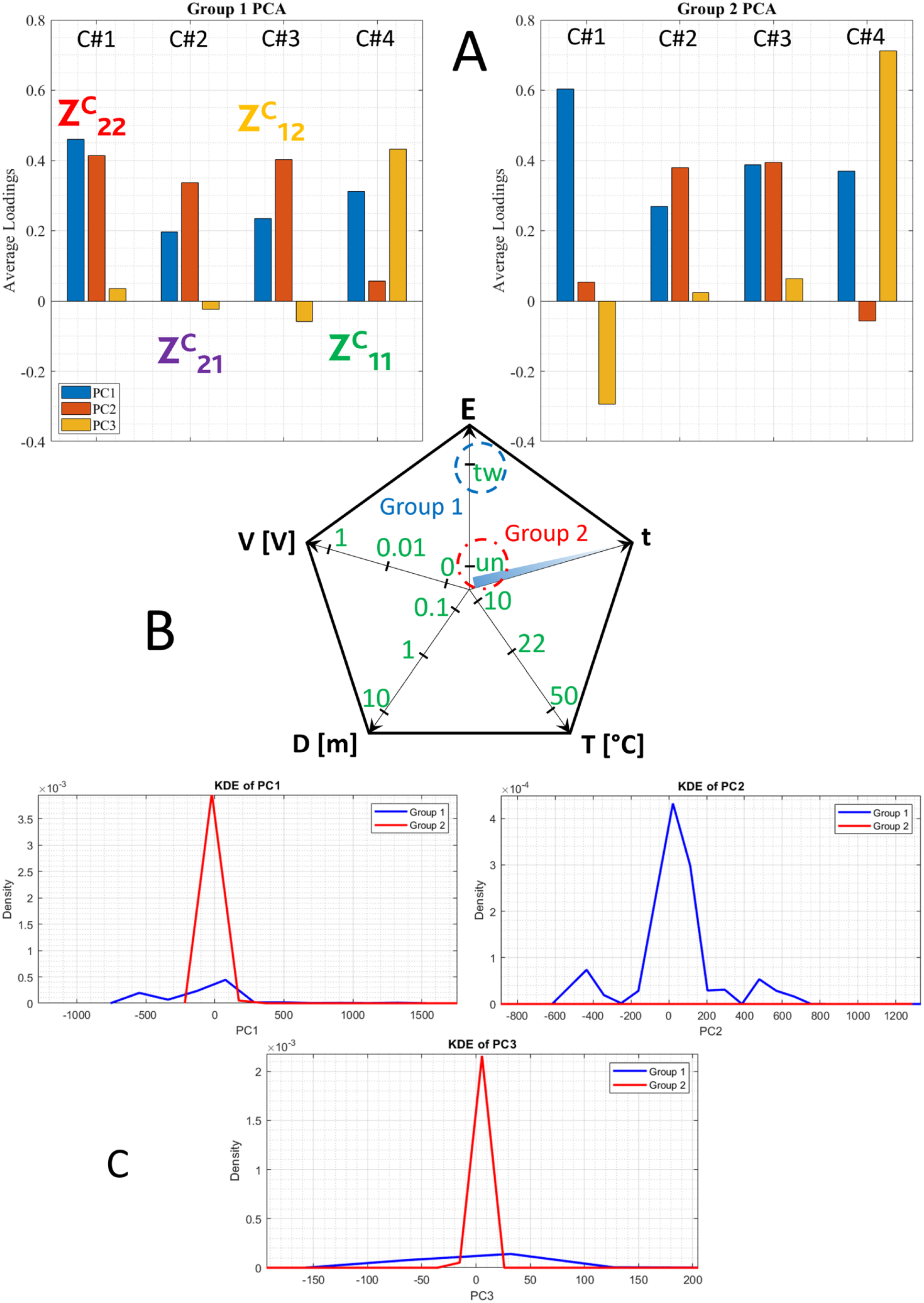
Average PCA loadings for the two experiment groups under comparison: twinned versus untwinned scattering components (**A**); radargram of the explored physical parameters space (**B**); Kernel Density Estimate (KDE) for each principal component in the twinned (group 1) and untwinned (group 2) pre-conditioning (**C**). The statistical sample is composed of 4.32 million entries for each of the two groups under comparison.

Figure 8 summarizes other relevant results collected by ML tools in comparing the two sets of experiments, when twinning pre-conditioning is performed (referred to group 1) with the case with no pre-conditioning at all (referred to group 2). Average PCA loadings can be used to qualitatively compare the two pre-conditioning states (Fig. 8A), twinned versus untwinned. The term “C#n” on the x-axis of average PCA loadings plot refers to the original variables in our dataset: C#1 is the reflection component $Z_{(2,2)}$ (red label), C#2 is the transmission component $Z_{(2,1)}$ (purple label), C#3 is the transmission component $Z_{(1,2)}$ (yellow label) and C#4 is the reflection component $Z_{(1,1)}$ (green label). Each bar in the plot corresponds to one of these original features. PCA loadings (coefficients) represent the weight or contribution of each original feature to the Principal Component (PC). A loading plot displays these weights for each PC. A positive loading indicates that the feature and the PC change in the same direction. As the feature value increases, the PC also increases. A negative loading indicates that the feature and the PC change in opposite directions. As the feature value increases, the PC decreases.

We could evaluate if there are sign changes in any of the three PCs or also differences in predominance (we look for the higher PC). Here, we notice 4 sign changes (3 featuring minor changes because of small amplitude, but feature 1) and 4 predominance changes (3 related to feature 1). We might say that the reflection feature 1 has strongly changed when passing from the twinned to the untwinned case.

Let us now introduce the radargram of Fig. 8, panel (B): it shows the explored parameters space, starting from the vertical axis **E** in a counter clock-wise direction: **E** is the entanglement state, either **tw** twinned or **un** untwinned; **V** is the DC stimulus amplitude: 1 V, 10 mV, 0 V; **D** is the distance between vials: 10 cm, 1 m, 10 m; **T** is the experiment temperature: $10\,^{\circ }$C, $22\,^{\circ }$C, $50\,^{\circ }$C; **t** is the natural time, since the sample is always the same, it ages linearly.

During every machine-powered run, Group 1 (blue) is compared to Group 2 (red). In this specific case, we have compared all experiments performed after “twinning” pre-conditioning with all experiments performed in the “untwinned” state. When a parameter is shown in green, it pertains to the group under study. When it is in black, it is excluded.

Further information comes from the Aggregated PCA Scores (APCAS). As our analysis encompasses a PC space having three dimensions, we end up with three bidimensional diagrams showing their inter-dependencies. The Kernel Density Estimate (KDE) provides a density measure of said inter-dependencies, shown in the form of a continuous curve (Fig. 8C). In this analysis, 2 out of 3 graphs show that the untwinned case produces denser and less broad distributions.

**Fig. 9:**
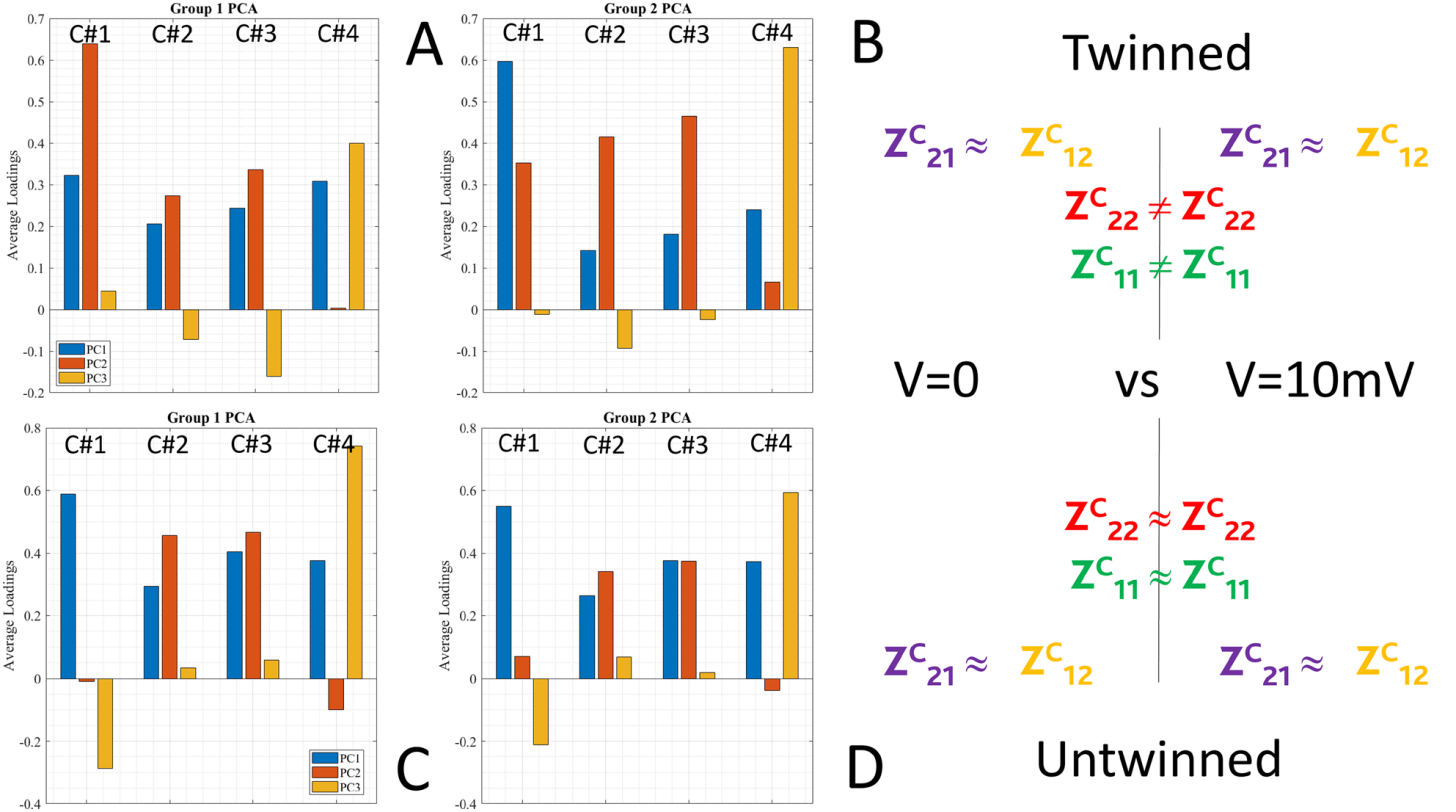
Effects on the two pre-conditioning states induced by the applied DC bias. Twinned case: average PCA loadings for the two experiment groups under comparison: zero bias versus small positive bias (**A**); principal features summarized (**B**). Untwinned case: average PCA loadings for the two experiment groups under comparison: zero bias versus small positive bias (**C**); principal features summarized (D). The statistical sample comprises 288.000 entries for each of the two groups under comparison.

Let us now discuss the effects on the two pre-conditioning states induced by the applied DC bias (see Fig. 9).

The twinned case responds with a PCA featuring 1 minor sign change and 9 amplitude changes. The case at 0 V (group 1) has higher PC1 and more balanced features, while the case at 10 mV (group 2) has higher PC2 and more unbalanced features. Both reflection and transport components are quite different.

The untwinned case responds with 1 minor sign change and 2 minor amplitude changes, much less than the previous case. The case at 0 V (group 1) has slightly higher PC2 components, but both groups have a quite balanced situation. The bias signal does not affect the eventual changes of the impedance, as it would happen in a system where the two vials have no correlation. The effect of a small signal (10 mV) when compared to the null signal produces very different effects on the two populations: the twinned case features a symmetry in the transport components and a change in the reflection components, while the untwinned case remains unaltered and symmetrical.

## Further elements

### Null test

**Fig. 10:**
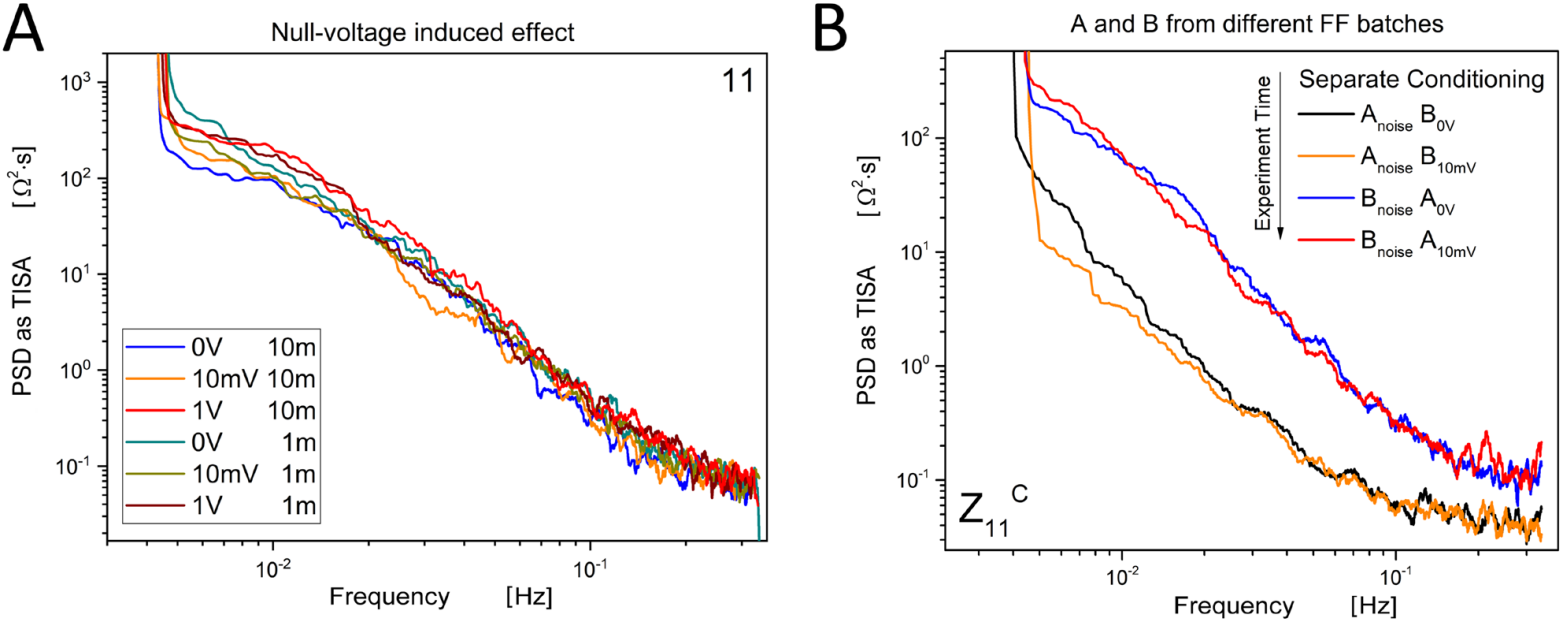
**A** Effects of a null-test on the two vials A and B: the DC generator was kept switched OFF during all the measurements, the scattering matrix reflection parameter Z_{1,1}_ is shown. The legend shows the different voltages applied and the distance between vials. The Power Spectral Density (PSD) of raw data fluctuations in the impedance “response” is plotted as TISA (Time-Integral Squared Amplitude) power, corresponding to the integral under the curve defined by the square of the raw data against time. The entire sample of 4.000 points is shown for each curve; an adjacent averaging smoothing over 51 points was used. (**B**): Effects of a pre-conditioning treatment performed separately on the two vials A and B: scattering matrix reflection parameter Z_{1,1}_. The legend shows the temporal order of execution: at first, the separate pre-conditioning was performed. Secondly, a run with vial A connected to the VNA and vial B submitted to 0 V applied. Third, the stimulus on vial B was raised to 10 mV. Fourth, vial B was connected to the VNA while vial A was submitted to 0V. Fifth, the stimulus on A was raised to 10 mV. The Power Spectral Density (PSD) of raw data fluctuations in the impedance “response” is plotted as TISA (Time-Integral Squared Amplitude) power, corresponding to the integral under the curve defined by the square of the raw data against time. The entire sample of 4.000 points is shown for each curve; an adjacent averaging smoothing over 101 points was used.

In this additional experiment, the DC generator was kept switched OFF. The results, shown in Fig. 10A, should be compared with those presented in Fig. 5B, the difference is remarkable.

### Pre-conditioning effect

Additional experiments were performed by submitting two vials to the same pre-conditioning hysteresis loops^53^ separately, and then verifying their phase correlation, according to the following conditions: 1 m distance, 0 V stimulus, and then 10 mV stimulus. The results are shown in the next Fig. 10B. We can see that the curves corresponding to vial A and those corresponding to vial B are almost overlapped and it is difficult to observe relevant differences among the two levels of stimulus applied.

### Divergence from uniform distribution

Relevant parameters are summarized in Table 1, including the Kullback-Leibler divergence, a metric distance between the experimental distributions under study and an ideal uniform distribution (zero means that the two distributions are identical).

**Table 1:** Relevant features of the cumulative statistics extrapolated from impedance measurements. $\sigma$ stands for standard deviation, “KLD uniform” is the Kullback-Leibler divergence calculated from an ideal uniform distribution.

Pre-conditioning	Mean	$\sigma$	Skewness	Kurtosis	Minimum	Maximum	KLD uniform
Twinned	−1.99	0.88	0.23737	0.13404	−3.92	0.13	1.759
Untwinned	−2.35	0.24	0.64189	0.0483	−2.73	−1.65	1.528

### Conjunct probability rule

We have compared the slope distributions shown in Fig. 6C and F, collecting the totality of experiments in the twinned and untwinned state, with the slope distributions of the subset of experiments featuring a voltage stimulus submitted to one of the vials and with the distributions of the subset of experiments featuring no voltage stimulus. If the system were classic, then classic conjunct probability rules would apply, namely:

10 $$\begin{aligned} P(A \cap B)= P(A) \cdot P(B|A) \end{aligned}$$

Now let us define $P(A \cap B)$ the probability that $Z_{1,1}^{C}(A)$ would deviate in the case *B* has been submitted to a voltage stimulus, *P*(*A*) as the probability that $Z_{1,1}^{C}(A)$ would deviate, and *P*(*B*) as the probability that *B* is submitted to a voltage stimulus (known to be exactly 17/28). Based on the measured data histogram distributions, we calculate that in the twinned case $P(A \cap B) = 56.25 \%$, and $P(A) \cdot P(B|A) = 28.76 \%$, therefore the joint probability rule does not apply. In the case of untwinned, non-pre-conditioned samples, the rule holds, as no deviation is measured, $P(A \cap B) = 0$ and also $P(A) = 0$. Similar situations hold for the other impedance scattering matrix reflection parameter $Z_{2,2}^{C}$ and transmission parameter $Z_{1,2}^{C}$. We believe this is strong supporting evidence of the non-classical nature of our effect.

### How quantum vibrational dipolar modes affect a ferrofluid

The composition of the EMG601P we have used comprises magnetite and water. Starting from the nominal magnetite weight fraction of the ferrofluid given by the manufacturer ($f^{w}_{m}=$0.2602), we should derive the magnetite volume fraction $f^{v}_{m}$. To do so, we calculate the volume occupied by magnetite in 1 g of ferrofluid i.e. $f^{w}_{m}/ \rho _{m}$ ($\rho _{m}$=5.18 $g/cm^{3}$^54^) equal to 0.05 $cm^{3}/g$; water occupies the balance ($f^{w}_{sol}=$1 − 0.2602=0.7408) whose density as known is 1 $g/cm^{3}$, gives also their volume. The magnetite volume fraction $f^{v}_{m}$ is derived using a simple proportion and is equal to 0.0633. On the basis of our experimental characterization of the EMG601P ferrofluid^51^, we find a weighted mean nanoparticle diameter of about $d_{NP}=$9 nm, corresponding to a mean particle volume of $V_{NP}=$382$~nm^{3}$. Using the magnetite unit-cell volume 0.6$~nm^{3}$^55^, this implies 637 unit cells per particle and therefore 2548 Bohr magnetons per particle (4 per unit cell^56^). The ratio between the mean particle volume and the magnetite volume fraction gives us the mean solvent volume available for each particle $V_{NP}/f^{v}_{m}=V_{sol}$, a cube of 6035$~nm^{3}$, whose side is therefore 18.21 nm and is also equal to the average center-to-center particle distance. Considering the nanoparticle mean diameter $d_{NP}$, this yields an average inter-particle separation of $d_{inter}=$9.21 nm. Defining the characteristic spacing between magnetic moments based on this experimentally determined volume per particle, we have $d_{eff} = (V_{eff}/N_{\mu })^{1/3} = (V_{NP}+V_{sol}/N_{\mu })^{1/3} = (6417 / 2548)^{1/3} \approx 1.36$, with $N_{\mu } =$ 2548 Bohr magnetons per particle. The de Broglie wavelength is given by $\lambda = h/p$, where *h* is the Planck constant ($6.626 \cdot 10^{-34}$ Js) and *p* is the momentum. At the equilibrium, $p=\sqrt{3 m_{e} n_{\mu B} k_{B} T}$: here $m_{e}$ is the mass of the electron ($9.109 \cdot 10^{-31}$ kg), $k_{B}$ the Boltzmann constant ($1,38 \cdot 10^{-23}$ J/K and *T* the temperature, in our calculus we set $T=323.15$ K). We thus obtain $\lambda = h/p= 3 ~nm > d_{eff}=1.36 ~nm$ and $\lambda ^{3} / d_{eff}^{3} = 10.73 > 1$. This result shows that, according to the standard criterion^57,58^, the particles with magnetic moments within each nanoparticle are in the quantum regime up to the temperature of $T=323.15$ K. The magnetic moment carried by them, in continuous Brownian motion, has a nonzero $\nabla \times$ **B** generating, according to Maxwell equations, a variation in time of the polarisation **P**. This last one is generated by the quantum vibrational dipolar modes of the bath of water and surfactant molecules, in which the magnetite nanoparticles are suspended. By simple calculations, it is possible to show that each magnetite nanoparticle, carrying 2548 Bohr magnetons, is surrounded by a sea of $\approx 2 \times 10^{5}$ electrical dipoles, without considering the contribution coming from the surfactant molecules. Therefore, also in such an approximation, the electronic cloud around magnetite is deeply affected by said quantum vibrational dipolar modes, suggesting us how the quantum effects can affect the interparticle correlations.

### Interference tests

**Fig. 11:**
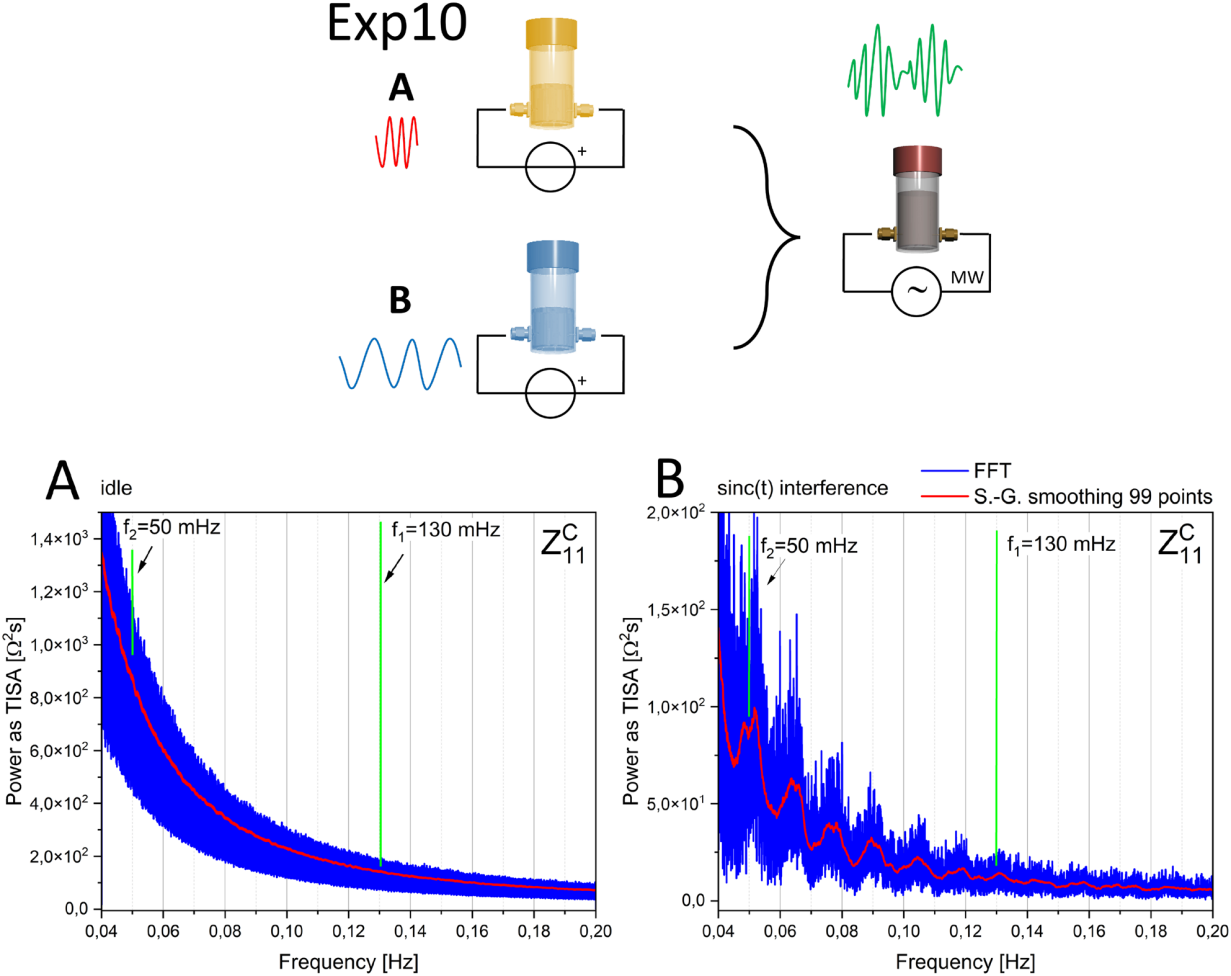
Top: conceptual sketch illustrating Exp10. Two separate vials containing 2 mL of FF each are submitted to a periodic stimulus, identical in amplitude but featuring slightly different frequencies, for 3 hours. Subsequently, the liquids are joined in a single vial and their impedance is characterized. The green waveform suggests that interference is observed, as in genuine phase phenomena. Bottom, panel A: Effects of the interference test on 4 mL of FF measured in a single vial without being submitted to any stimuli. B: the vial where the two aliquots have been joined after being submitted to the two different waveforms. In both panels, the scattering matrix reflection parameter Z_{1,1}_ is shown. The Power Spectral Density (PSD) of raw data fluctuations in the impedance “response” is plotted as TISA (Time-Integral Squared Amplitude) power, corresponding to the integral under the curve defined by the square of the raw data against time. The entire sample consists of 32.000 points; an adjacent averaging smoothing over 99 points was used. Green lines indicate the frequencies of the two stimuli and the expected beat frequency, which actually corresponds to an observable peak.

We verified the presence of interference in another set of experiments, sketched in Fig. 11. Two vials, filled with 2 mL of FF each, are submitted to a periodic stimulus, a *sin*(*t*)/*t* waveform, with peak-to-peak amplitude 1 V, offset -250 mV, and frequencies equal to 50 (vial A) and 130 (vial B) mHz, for 3 hours. Then the liquids are joined in the same vial A, and their impedance is measured when no stimulus is applied. The characteristic beat peak appears at a frequency corresponding to the difference between the two pristine signals. The measurements are repeated to increase the statistical significance of impedance fluctuations until 32.000 points are collected (approximately 14 hours of measurement time).

## Conclusions

In conclusion, we have reported the experimental observation of statistically relevant phase correlations between separated aliquots of two different commercial ferrofluids (FFs), after both underwent a specific pre-conditioning (“twinning”) and were subsequently subjected to voltage stimuli. In both cases, impedance measurements were acquired over a wide frequency spectrum and within a controlled temperature range of 10–50 °C, ensuring that the FFs retained their liquid characteristics throughout the experiments. Under these conditions, we observed phase correlations that persisted after physical separation of the aliquots and in the presence of electromagnetic shielding, so that direct electromagnetic coupling between the two vials can reasonably be excluded within the sensitivity and design limits of our setup. Our experimental strategy was explicitly constructed from a null-hypothesis standpoint, progressively testing and ruling out a series of classical coupling mechanisms compatible with our geometry and instrumentation, and quantifying the remaining correlations using impedance-based features and multivariate analyses. Within this framework, the data show a reproducible conditional relationship between the electrical response of the twinned ferrofluid vials under the specific pre-conditioning and measurement protocol described. At the same time, important limitations must be emphasized: we tested only two proprietary ferrofluids, one custom vial design and one electronic architecture, over finite distances and a restricted temperature range; we cannot conclusively exclude all possible hidden couplings (e.g. residual mechanical micro-vibrations induced by the building); and our impedance analysis relies on feature extraction and statistical modeling steps that may introduce biases. Future work should focus on blinded and independent replication, systematic variation of particle sizes, shapes and carrier liquids, alternative hardware implementations and more stringent isolation tests, before drawing conclusions about the generality of the observed phase correlations.

## Supplementary Information

Supplementary Information.

## Data Availability

Data are available here: https://zenodo.org/records/14739812. Chiolerio, A. (2025). Room Temperature Colloidal Entanglement in Ferrofluids [Data set]. Zenodo. https://doi.org/10.13140/RG.2.2.14629.7216
