# Supplementary material for: Observation of phase correlations in ferrofluids
Source: Sci Rep. 2026 Apr 5;16:16510. doi: 10.1038/s41598-026-45834-1 (PMC13216242; doi:10.1038/s41598-026-45834-1)
Supplement: Supplementary file 1 — Supplementary Information. [file 41598_2026_45834_MOESM1_ESM.pdf]

Observation of phase correlations in ferrofluids – Supplementary Information

Alessandro Chiolerio[1,2,*], Giuseppe Vitiello[3], Mohammad Mahdi Dehshibi[4], Marco Crepaldi[5], Diego Torazza[6], and Andrew Adamatzky[2]

[1]Bioinspired Soft Robotics, Istituto Italiano di Tecnologia, Via Morego 30, Genova, 16163, Italy
[2]Unconventional Computing Laboratory, University of the West of England, Coldharbour Lane, Bristol, BS16 1QY, England, UK
[3]Dipartimento di Fisica "E.R. Caianiello", Universitá di Salerno, Via Giovanni Paolo II 132, Fisciano, 84084, Italy
[4]Department of Computer Science and Engineering, Universidad Carlos III de Madrid, Avda. de la Universidad 30, Leganès, 28911, Spain
[5]Electronic Design Laboratory, Istituto Italiano di Tecnologia, Via Melen 83, Genova, 16152, Italy
[6]Mechanical Workshop, Istituto Italiano di Tecnologia, Via S. Quirico 19d, Genova, 16163, Italy

ABSTRACT

This document contains the supplementary information for the manuscript entitled "Observation of phase correlations in ferrofluids".

Contents:

About memory mechanisms in colloids 1
Measurements 2
Machine-Powered Analyses 3
Distance Effect 4
Temperature Effect 5
Time Effect 6
Volume Effect 7
Solvent Effect 8
Relaxation Time and Electrochemical Effects9

1 About memory mechanisms in colloids

By "memory of the colloidal arrangements" we mean slow, history-dependent structural states of the suspension: different topologies, local volume fraction gradients, ionic layers near electrodes, and the associated hydrodynamic and electrical responses that relax on long timescales. Therefore, such a memory is a structural, hydrodynamic, and electrostatic memory, not based on remanence. In concentrated fluids, electric dipolar coupling can produce mesoscopic aggregates with slow magneto-structural relaxation. Those slow modes can contribute to the observed impedance memory. The paper by Singh et al.[1] remarkably offers an excellent clue to understanding the memory effects observed in ferrofluids, in terms of spin glasses: "The highly interacting nanomagnetic systems, especially the single-domain ones are observed to show various magnetic configurations in DC magnetization (at low fields) and AC susceptibility measurements for magnetic relaxation (at low frequencies) because of various competing energies. [...] The memory effects in the magnetic phases suggest the arrangement of metastable states below the blocking temperature analogous to spin glass behaviour." Practically, the relaxation we observe reflects a combination of mechanisms: structural (aggregate breakup / reformation, steric constraints), interfacial (double-layer re-equilibration at electrodes), and magnetic (dipolar couplings that modulate structure). For individual particles, magnetic moment relaxation occurs by Néel flipping ($\tau_N \approx \tau_0 exp(KV/k_B T)$ where K is the magnetic anisotropy energy density, V is the volume of the particle, tau_0 is the attempt time, k_B is the Boltzmann constant and T is the temperature) and/or Brownian rotation ($\tau_B \approx 3\eta V_h/k_B T$ where η is the dynamical viscosity, and V_h is the hydrodynamic volume). In a highly concentrated ferrofluid with elevated viscosity and interparticle constraints, Brownian rotation is hindered, pushing the balance toward Néel processes for smaller particles and toward collective, cluster-level rearrangements for larger aggregates. This aligns with our prior note that the usual low-frequency "Brownian peak" can be suppressed in such systems.

As mentioned in Sec. 1 of the text, the formation of ordered domains is described in terms of the many-body physics in quantum field theory (QFT)[2,3]. The spontaneous breakdown of the symmetry (SBS) of the time evolution of the system components, which is the spherical rotational symmetry in the case of the FF electrical and magnetic dipoles, produces long-range phase correlations (the Goldstone theorem in QFT[2–5]) binding them into ordered coherent domains. One central dynamical feature is the appearance of the entanglement among the phase correlated dipoles[6]. Memory effects in highly correlated many-body systems arise when ergodic mixing is hindered, so that information about initial conditions persists in observables over times much longer than expected. In disordered, interacting lattices, Many-Body Localization (MBL) provides a paradigmatic mechanism: emergent quasi-local motion arrests thermalization, preserving the local density patterns and the entanglement structure[7]. In frustrated magnets, spin-glass landscapes encode memory via hierarchical metastable valleys and large energy barriers, producing ageing, rejuvenation and temperature-cycling memory in magnetic susceptibilities[8]. Spatially, these memories are typically quasi-local (MBL integrals of motion) and domain-based (spin glasses). They are destroyed by channels that restore ergodicity, such as environmental coupling, heating under strong periodic drive, or reduction of disorder / raising energy density, thereby erasing the stored correlations. In the main text, we have discussed details of such a phenomenon (see in particular Section 6).

2 Measurements

In the following figures, we show the bi-logarithmic plots of the parameter Z_{11}^C for the entire set of experiments of both the untwinned and twinned groups. The statistical sample shown in every graph is composed by 4.000 entries for each of the plotted curve. Unspecified units of measures, from here till the end of the document, are $\Omega^2 \cdot s$ for the Power as TISA (y-axis) and Hz for the Frequency (x-axis).

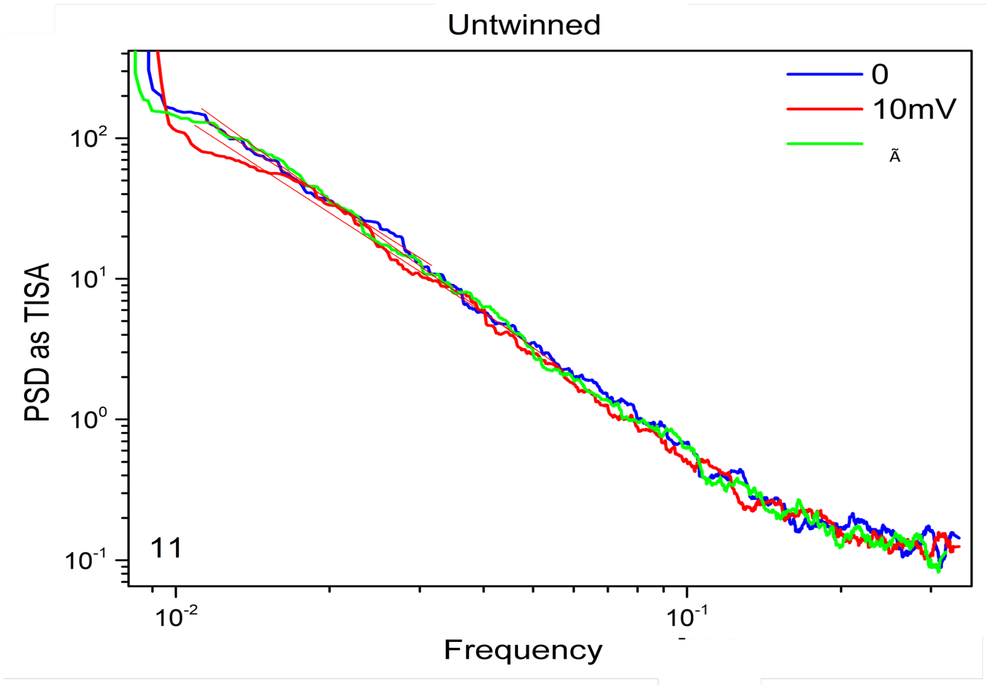

Figure 1. Z_{11}^{C} fluctuations during Exp1 in the untwinned case.

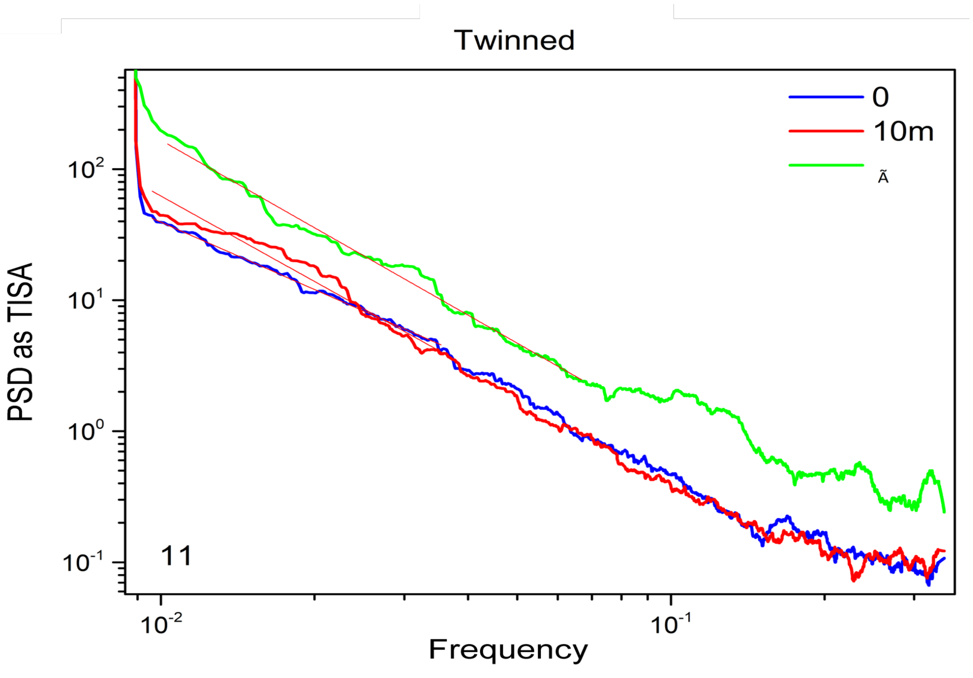

Figure 2. Z_{11}^{C} fluctuations during Exp1 in the twinned case.

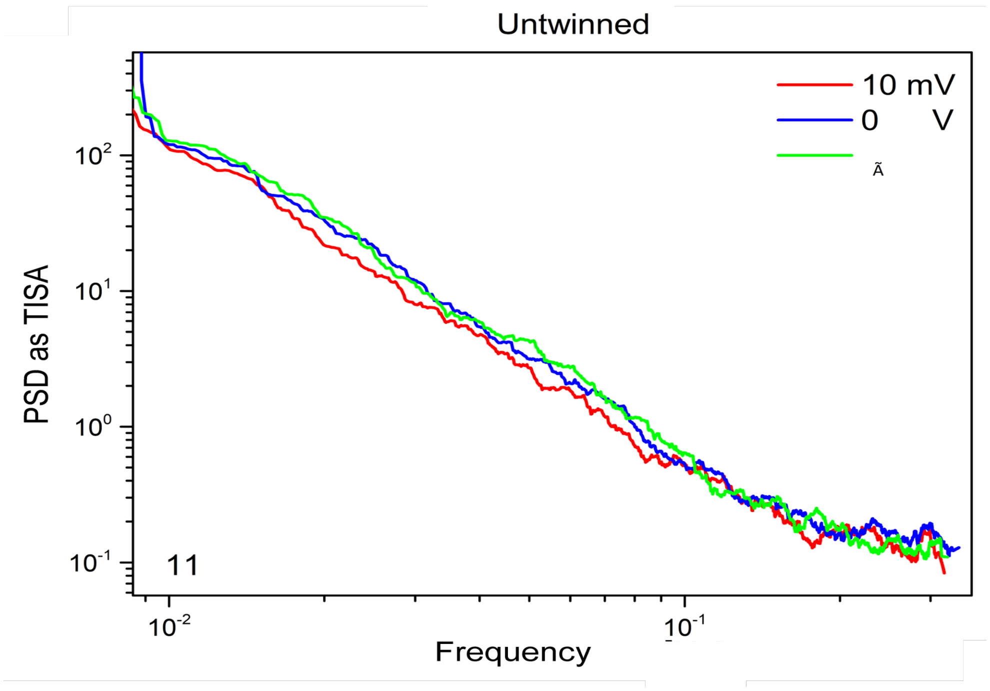

Figure 3. Z_{11}^{C} fluctuations during Exp2 in the untwinned case.

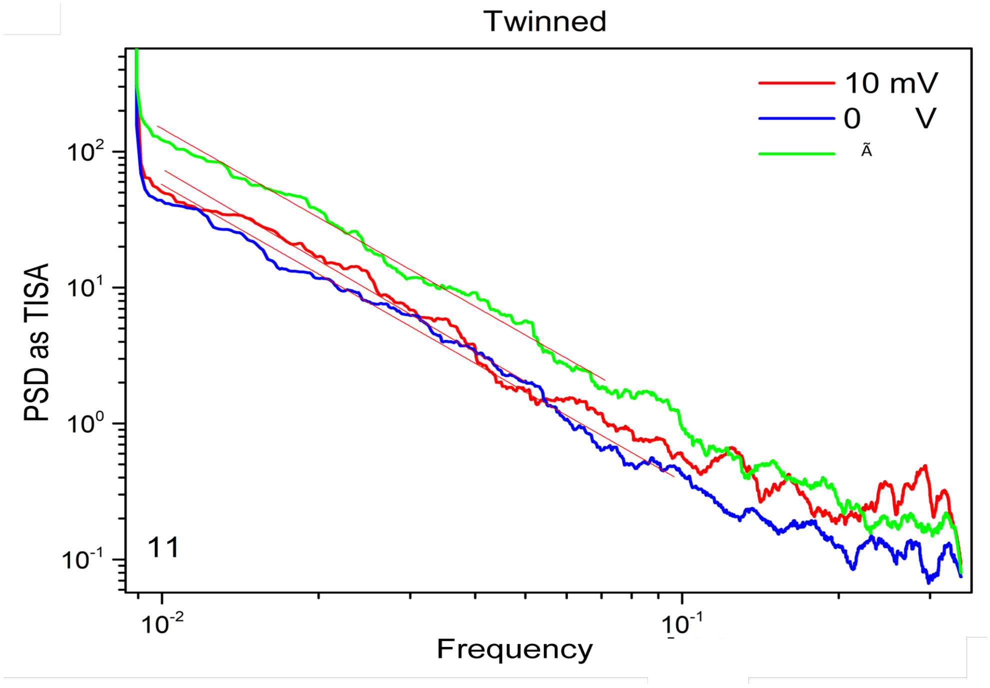

Figure 4. Z_{11}^{C} fluctuations during Exp2 in the twinned case.

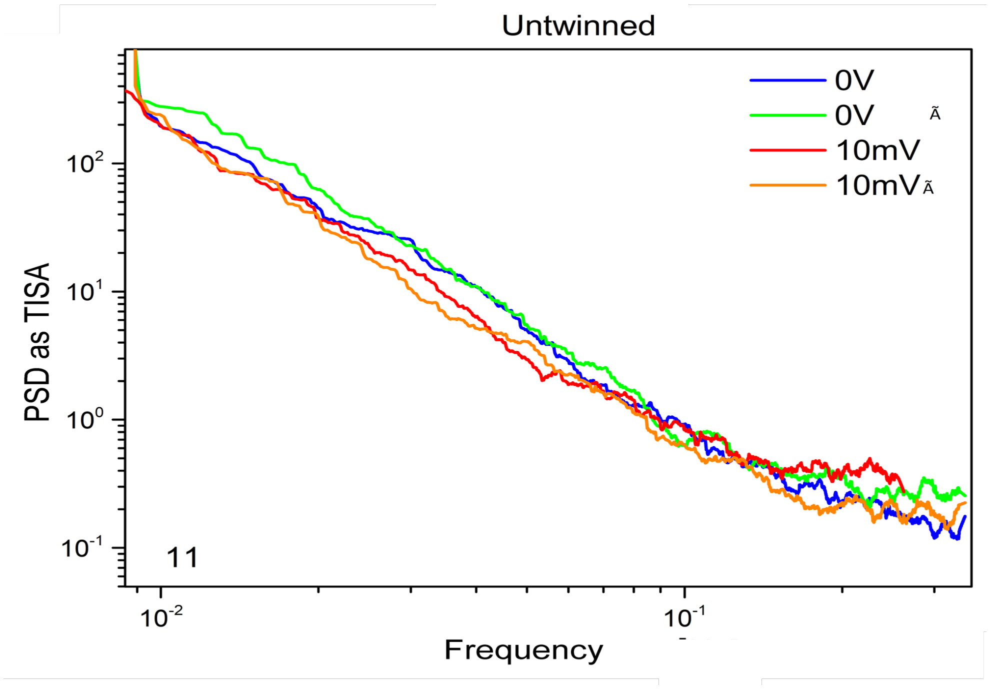

Figure 5. Z_{11}^{C} fluctuations during Exp3 in the untwinned case.

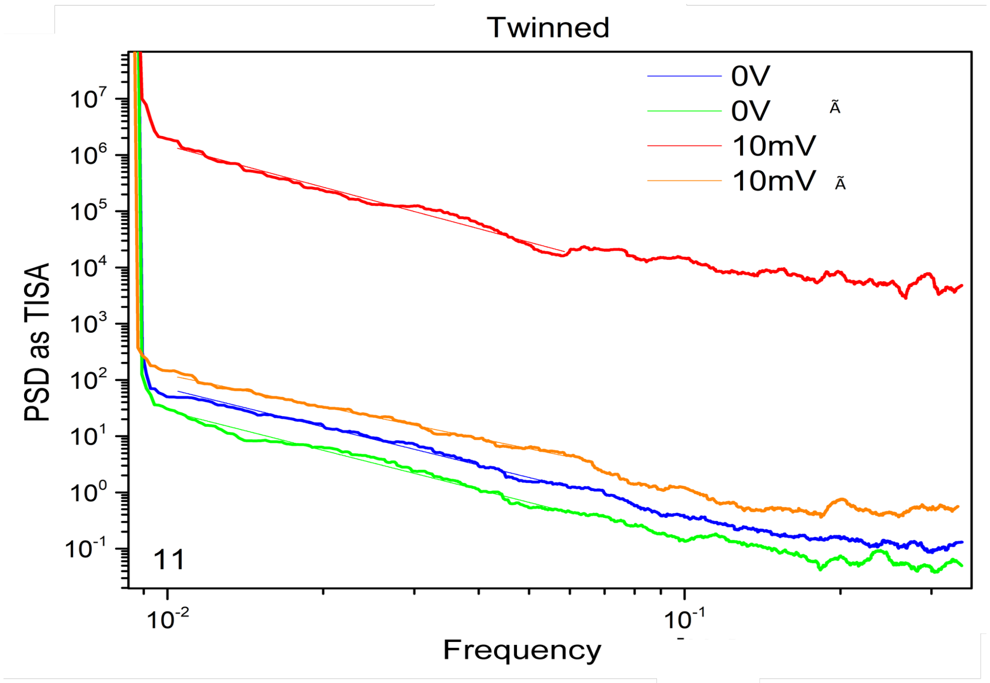

Figure 6. Z_{11}^{C} fluctuations during Exp3 in the twinned case.

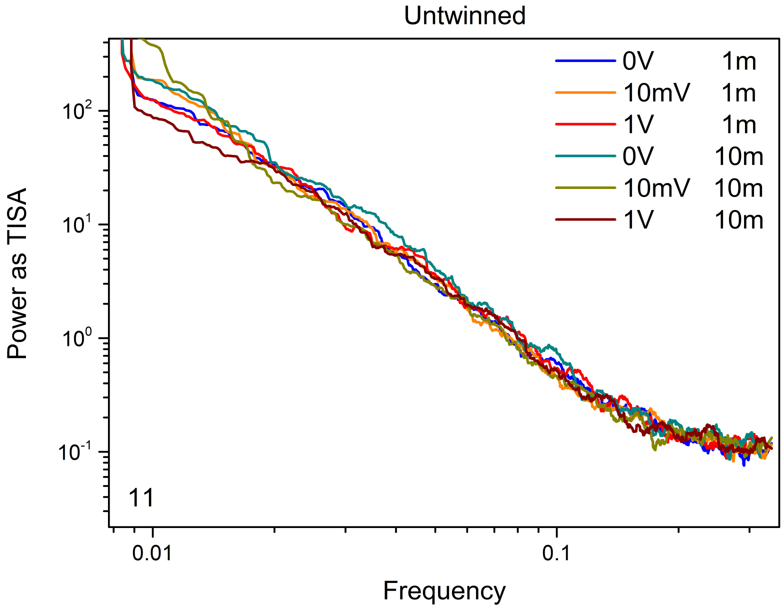

Figure 7. Z_{11}^{C} fluctuations during Exp4 in the untwinned case.

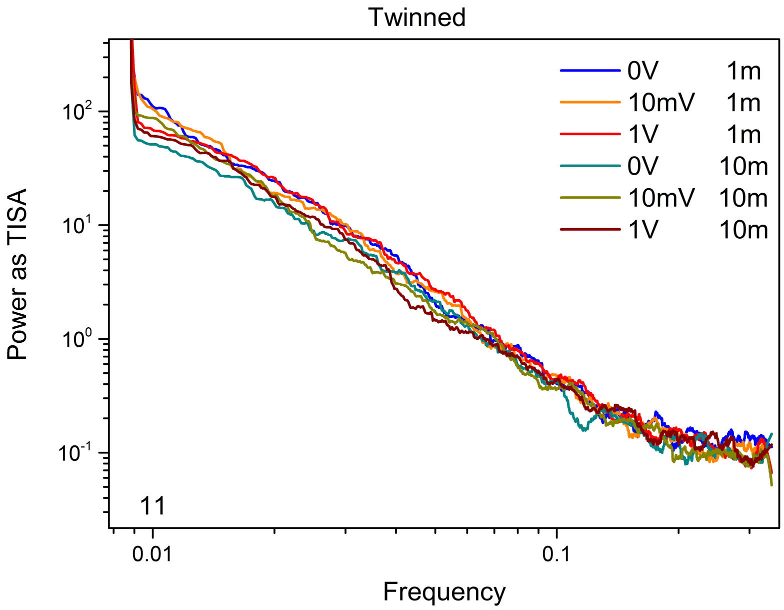

Figure 8. Z_{11}^{C} fluctuations during Exp4 in the twinned case.

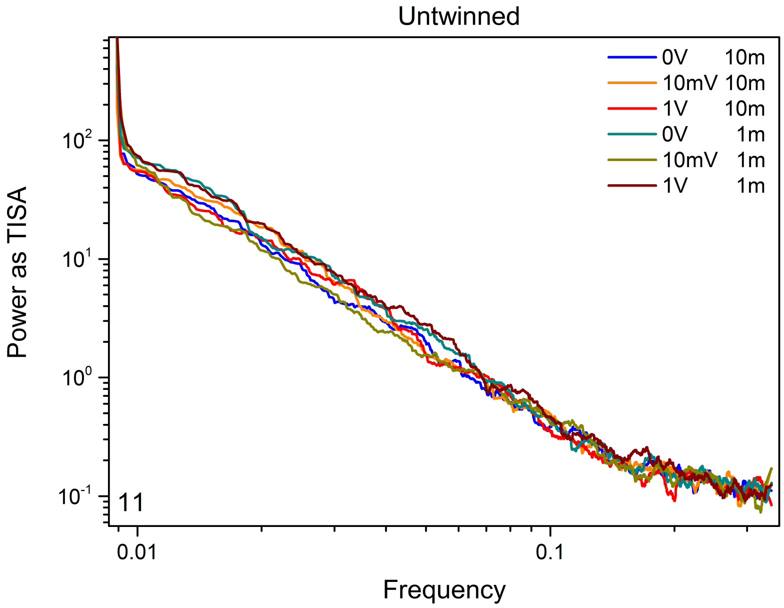

Figure 9. Z_{11}^{C} fluctuations during Exp5 in the untwinned case.

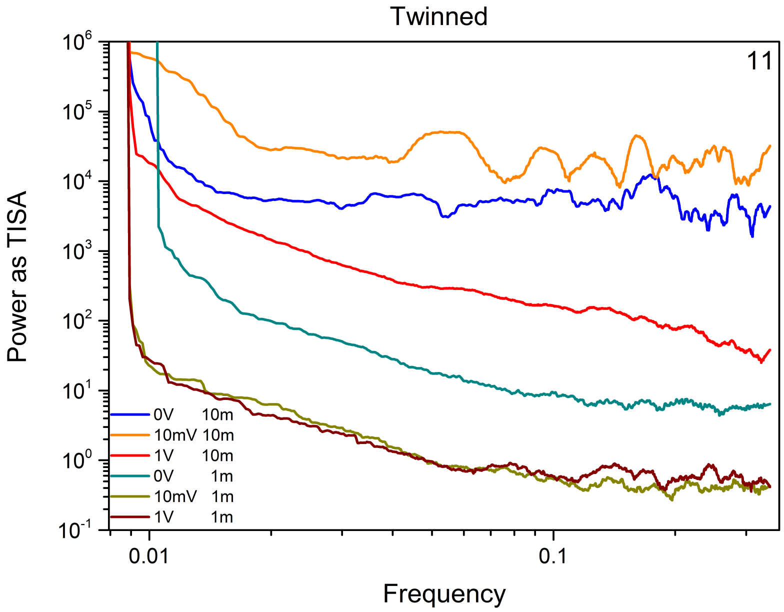

Figure 10. Z_{11}^{C} fluctuations during Exp5 in the twinned case.

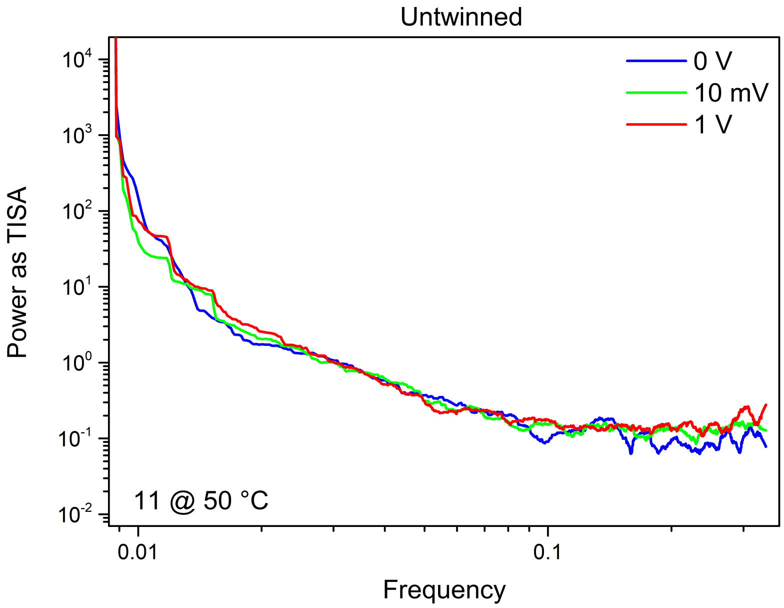

Figure 11. Z_{11}^{C} fluctuations during Exp6 in the untwinned case.

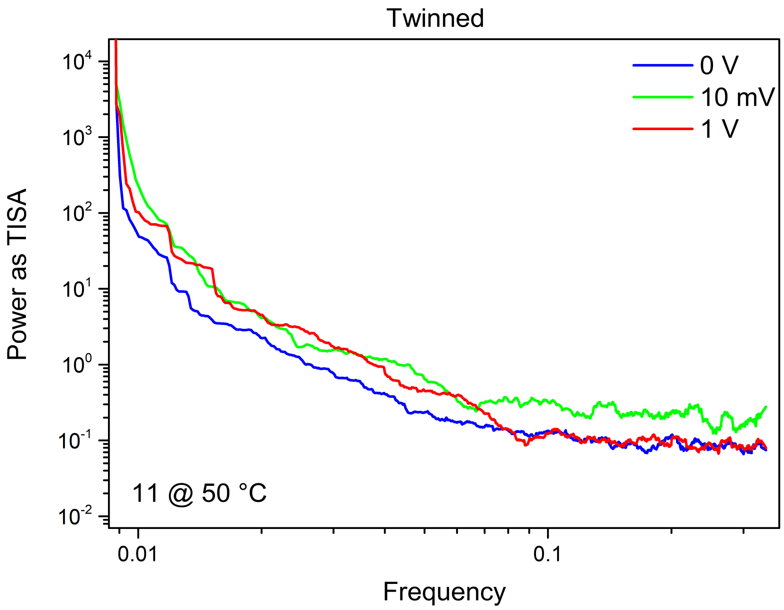

Figure 12. Z_{11}^{C} fluctuations during Exp6 in the twinned case.

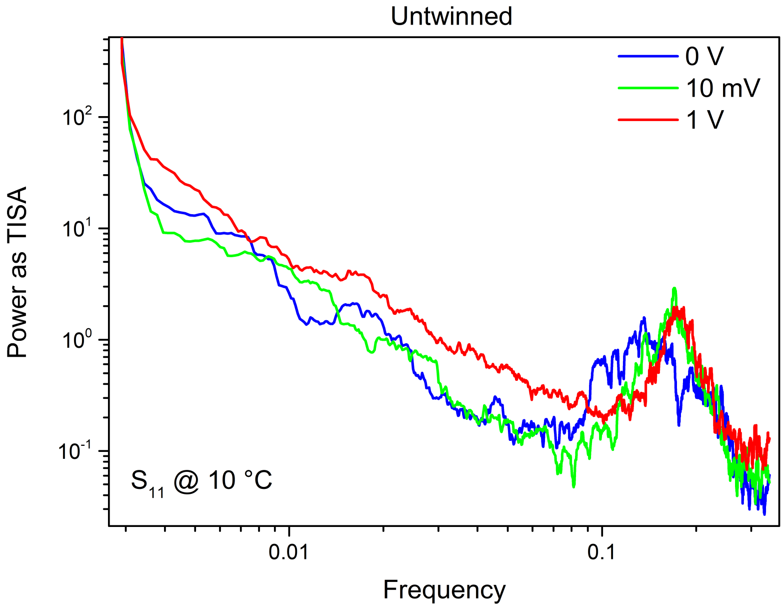

Figure 13. Z_{11}^C fluctuations during Exp7 in the untwinned case.

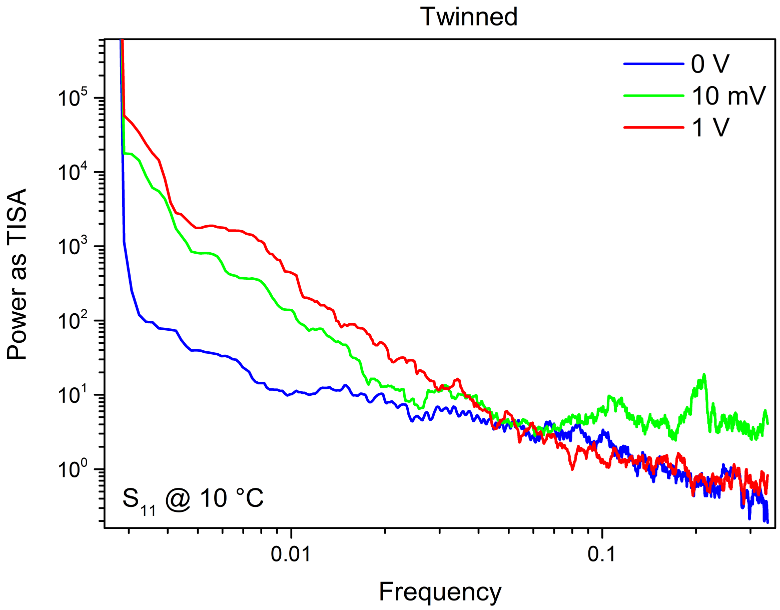

Figure 14. Z_{11}^C fluctuations during Exp7 in the twinned case.

3 Machine-Powered Analyses

In the following figures, we show the complete statistical machine-aided analysis of selected groups of experiments, as detailed in the captions. The radargram shown in panel (A) is conceived in this way: it shows all the parameters space, starting from the vertical axis **E** in a counter clock-wise direction: **E** is the entanglement state, either **tw** twinned or **un** untwinned; **V** is the DC stimulus amplitude: 1 V, 10 mV, 0 V; **D** is the distance between vials: 10 cm, 1 m, 10 m; **T** is the ambient temperature: 10°C, 22°C, 50°C; **t** is the natural time, since the sample is always the same, it ages linearly. During every comparison machine analysis, Group 1 (blue) is compared to Group 2 (red), in this specific case we have compared all cases under the twinned state with all cases under the untwinned state, with a subset of parameters indicated on the other axes. When a parameter is shown in green, it pertains to the group under study, when it is in black, it is excluded. The statistical sample size n for each group under comparison is indicated in the captions.

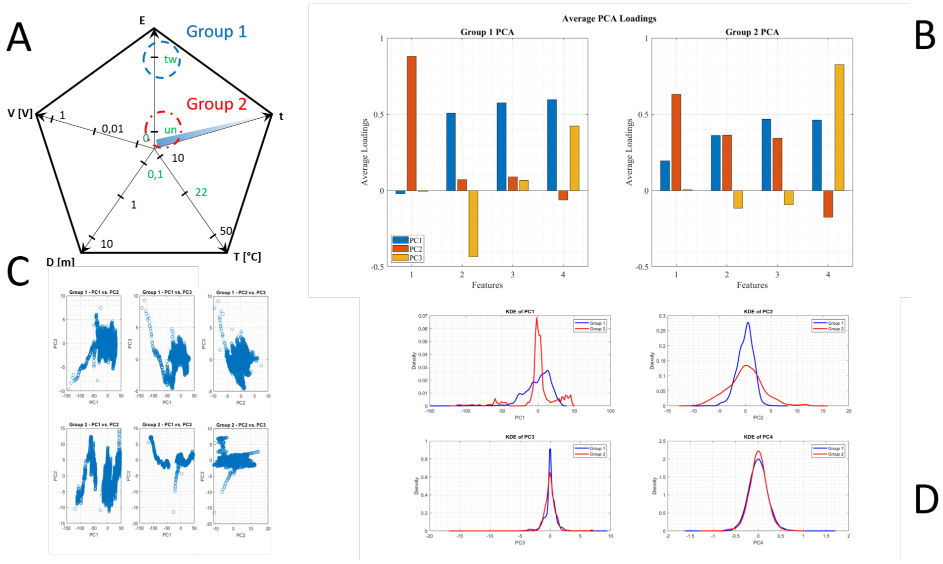

Figure 15. Machine-aided statistical analyses of the scattering matrix measurements involving the experiments performed with 0 V stimulus, at 10 cm distance, at 22 deg C, for the twinned versus untwinned cases. A: radargram depicting involved experiments over the entire experiment space. B: average PCA loadings of the two groups. C: aggregated PCA scores for the three principal components. D: kernel density estimates for the three principal components. $n = 48.000$.

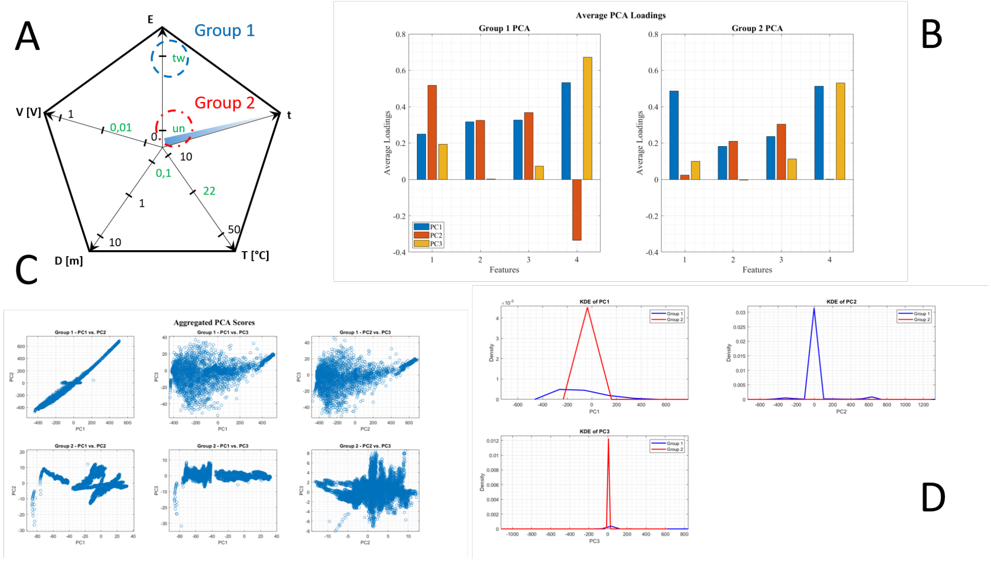

Figure 16. Machine-aided statistical analyses of the scattering matrix measurements involving the experiments performed with 10 mV stimulus, at 10 cm distance, at 22 deg C, for the twinned versus untwinned cases. A: radargram depicting involved experiments over the entire experiment space. B: average PCA loadings of the two groups. C: aggregated PCA scores for the three principal components. D: kernel density estimates for the three principal components. $n = 48.000$.

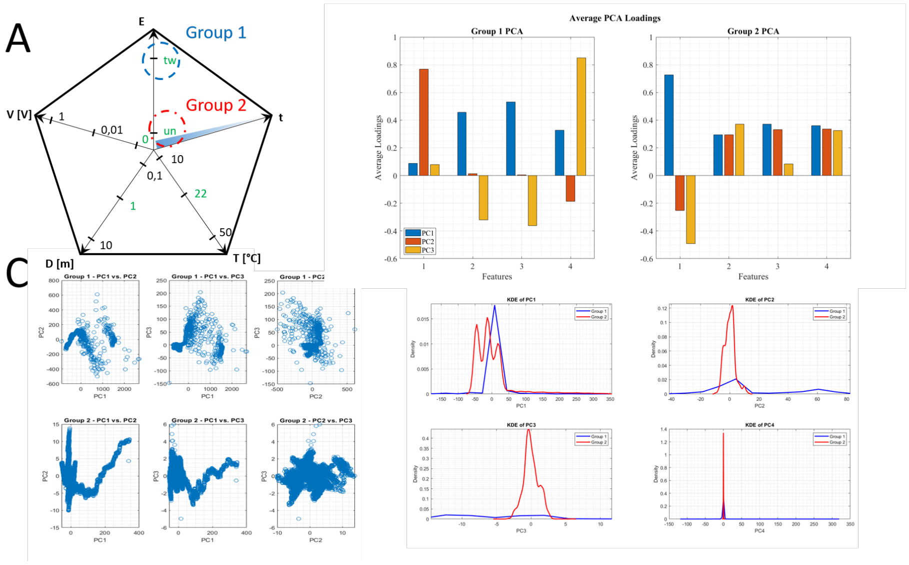

Figure 17. Machine-aided statistical analyses of the scattering matrix measurements involving the experiments performed with 0 V stimulus, at 1 m distance, at 22 deg C, for the twinned versus untwinned cases. A: radargram depicting involved experiments over the entire experiment space. B: average PCA loadings of the two groups. C: aggregated PCA scores for the three principal components. D: kernel density estimates for the three principal components. $n = 32.000$.

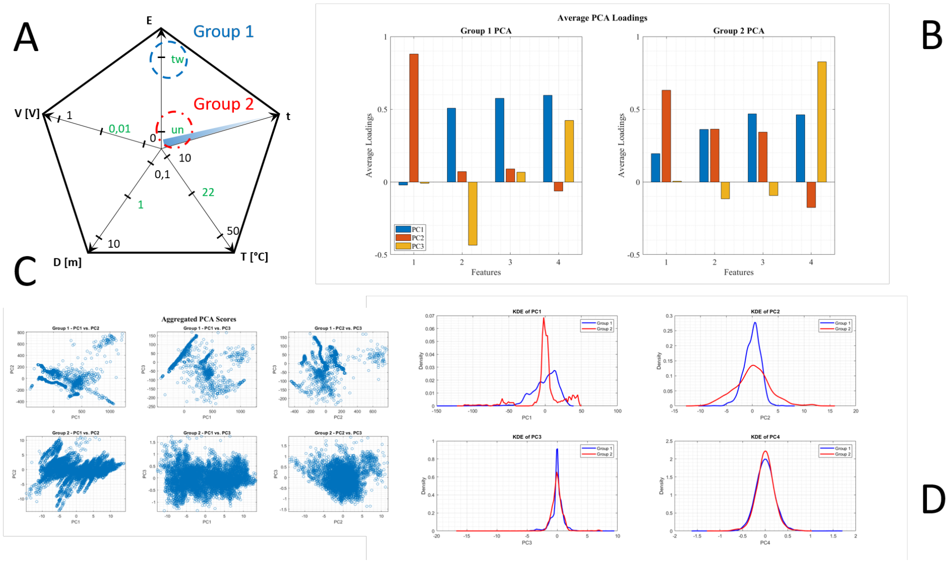

Figure 18. Machine-aided statistical analyses of the scattering matrix measurements involving the experiments performed with 10 mV stimulus, at 1 m distance, at 22 deg C, for the twinned versus untwinned cases. A: radargram depicting involved experiments over the entire experiment space. B: average PCA loadings of the two groups. C: aggregated PCA scores for the three principal components. D: kernel density estimates for the three principal components. $n = 32.000$.

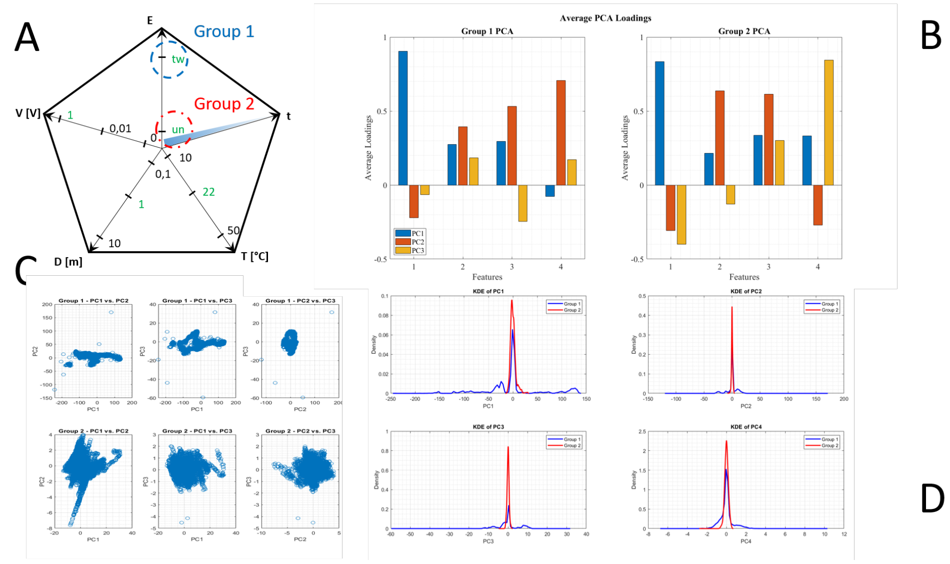

Figure 19. Machine-aided statistical analyses of the scattering matrix measurements involving the experiments performed with 1 V stimulus, at 1 m distance, at 22 deg C, for the twinned versus untwinned cases. A: radargram depicting involved experiments over the entire experiment space. B: average PCA loadings of the two groups. C: aggregated PCA scores for the three principal components. D: kernel density estimates for the three principal components. $n = 32.000$.

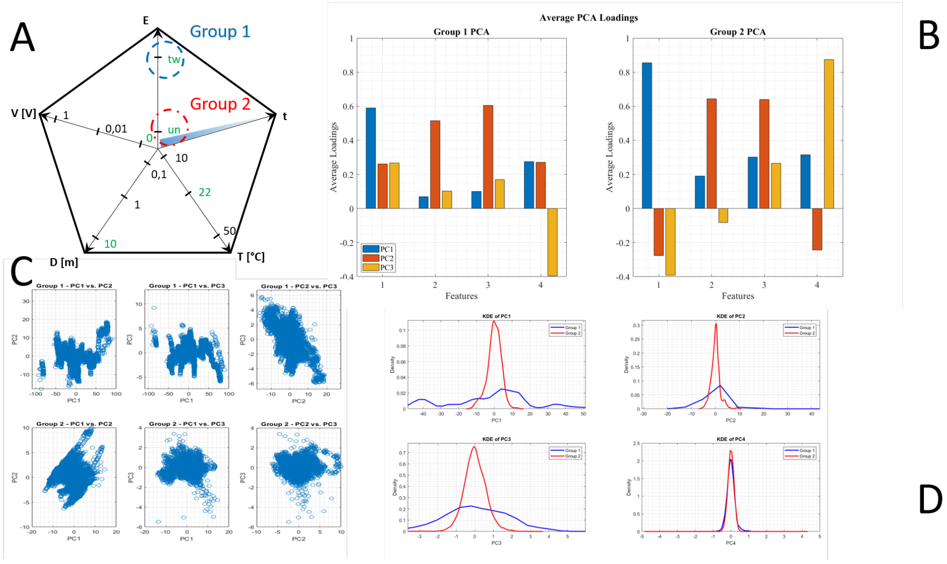

Figure 20. Machine-aided statistical analyses of the scattering matrix measurements involving the experiments performed with 0 V stimulus, at 10 m distance, at 22 deg C, for the twinned versus untwinned cases. A: radargram depicting involved experiments over the entire experiment space. B: average PCA loadings of the two groups. C: aggregated PCA scores for the three principal components. D: kernel density estimates for the three principal components. $n = 32.000$.

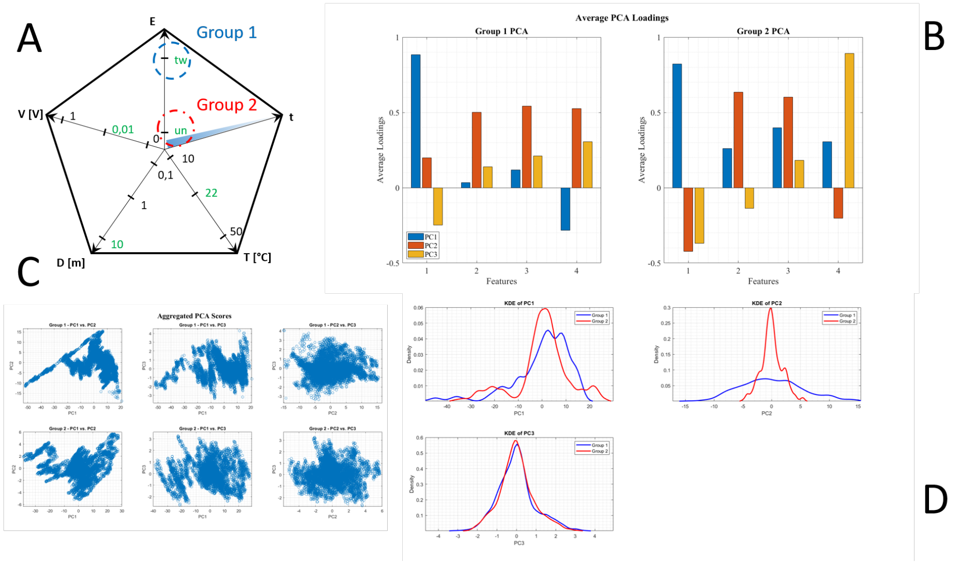

Figure 21. Machine-aided statistical analyses of the scattering matrix measurements involving the experiments performed with 10 mV stimulus, at 10 m distance, at 22 deg C, for the twinned versus untwinned cases. A: radargram depicting involved experiments over the entire experiment space. B: average PCA loadings of the two groups. C: aggregated PCA scores for the three principal components. D: kernel density estimates for the three principal components. $n = 32.000$.

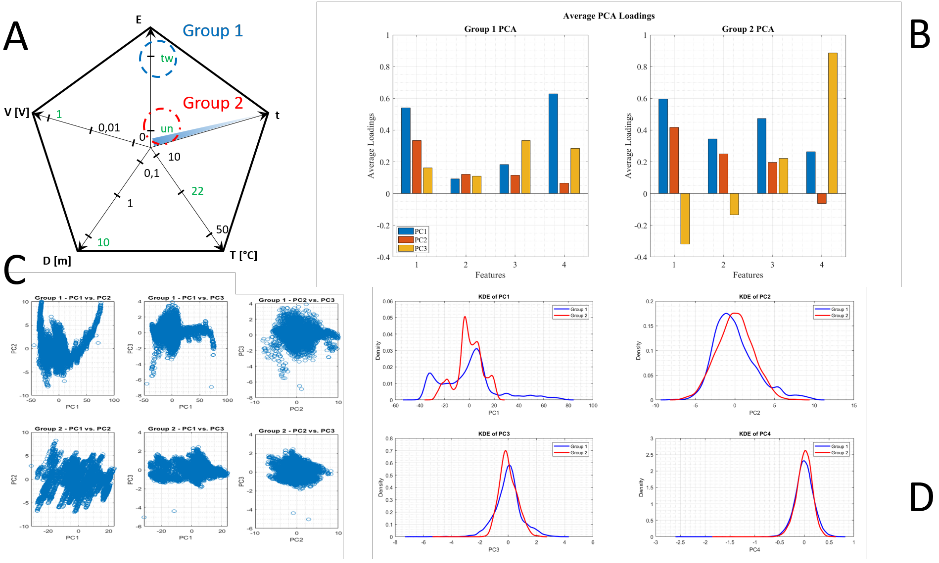

Figure 22. Machine-aided statistical analyses of the scattering matrix measurements involving the experiments performed with 1 V stimulus, at 10 m distance, at 22 deg C, for the twinned versus untwinned cases. A: radargram depicting involved experiments over the entire experiment space. B: average PCA loadings of the two groups. C: aggregated PCA scores for the three principal components. D: kernel density estimates for the three principal components. $n = 32.000$.

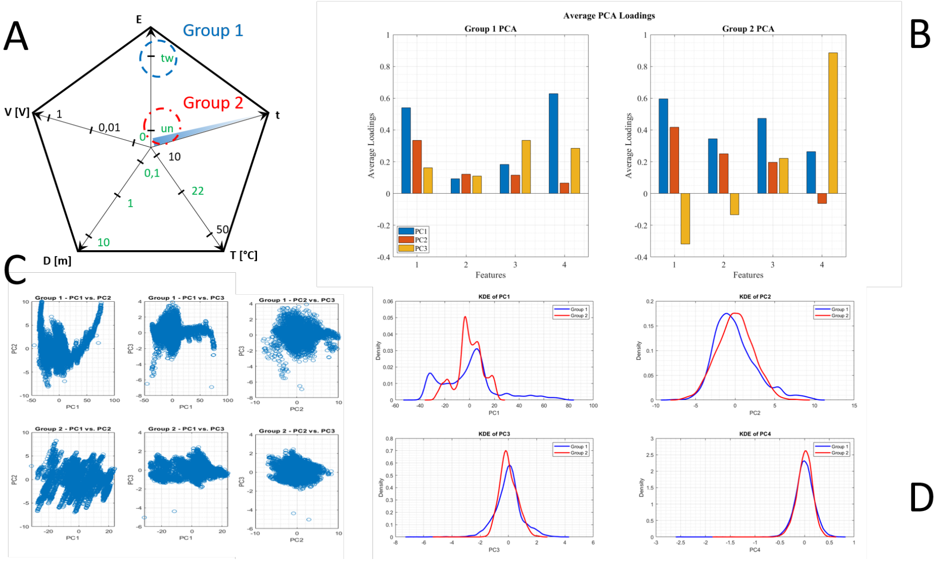

Figure 23. Machine-aided statistical analyses of the scattering matrix measurements involving the experiments performed with 0 V stimulus, at any distance, at 22 deg C, for the twinned versus untwinned cases. A: radargram depicting involved experiments over the entire experiment space. B: average PCA loadings of the two groups. C: aggregated PCA scores for the three principal components. D: kernel density estimates for the three principal components. $n = 84.000$.

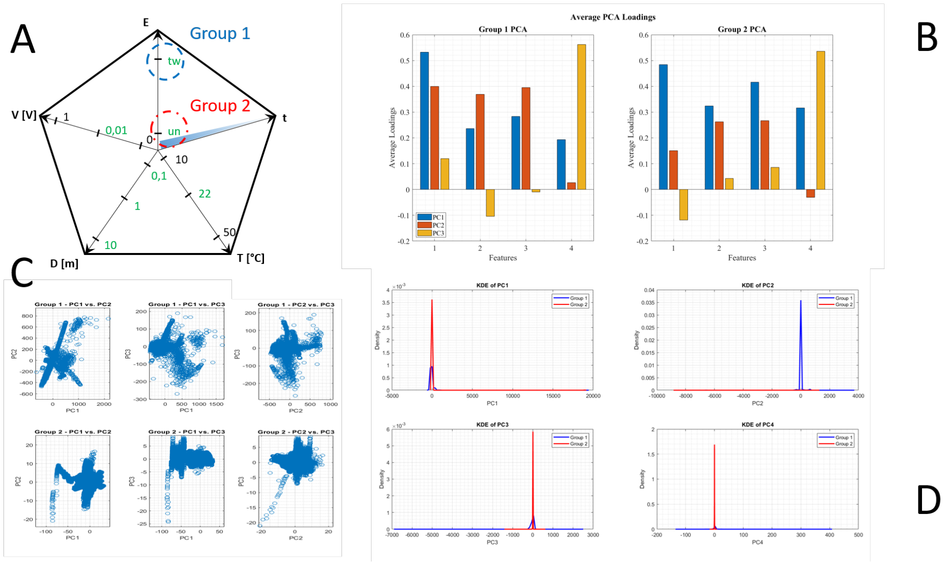

Figure 24. Machine-aided statistical analyses of the scattering matrix measurements involving the experiments performed with 10 mV stimulus, at any distance, at 22 deg C, for the twinned versus untwinned cases. A: radargram depicting involved experiments over the entire experiment space. B: average PCA loadings of the two groups. C: aggregated PCA scores for the three principal components. D: kernel density estimates for the three principal components. $n = 84.000$.

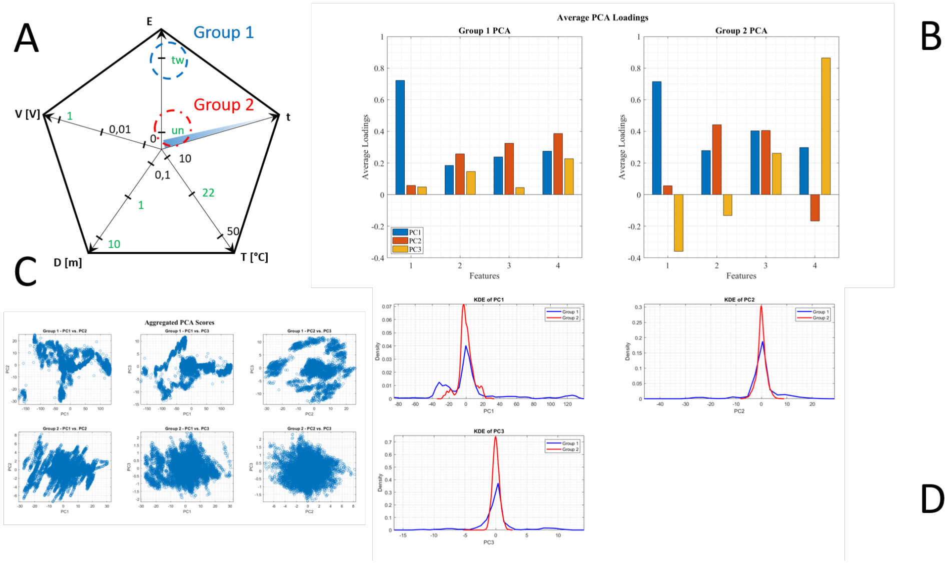

Figure 25. Machine-aided statistical analyses of the scattering matrix measurements involving the experiments performed with 1 V stimulus, at a distance of 1 or 10 m, at 22 deg C, for the twinned versus untwinned cases. A: radargram depicting involved experiments over the entire experiment space. B: average PCA loadings of the two groups. C: aggregated PCA scores for the three principal components. D: kernel density estimates for the three principal components. $n = 64.000$.

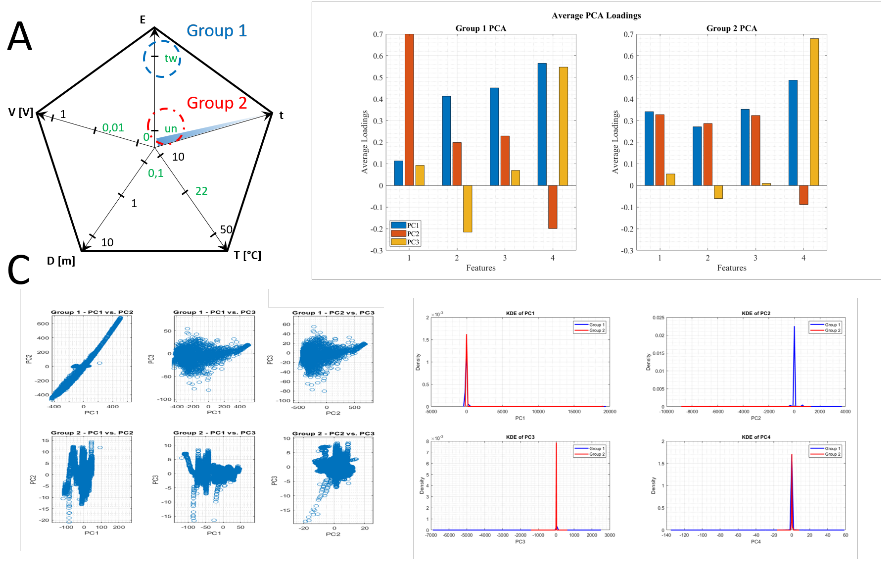

Figure 26. Machine-aided statistical analyses of the scattering matrix measurements involving the experiments performed with 0 or 10 mV stimulus, at a distance of 10 cm, at 22 deg C, for the twinned versus untwinned cases. A: radargram depicting involved experiments over the entire experiment space. B: average PCA loadings of the two groups. C: aggregated PCA scores for the three principal components. D: kernel density estimates for the three principal components. $n = 96.000$.

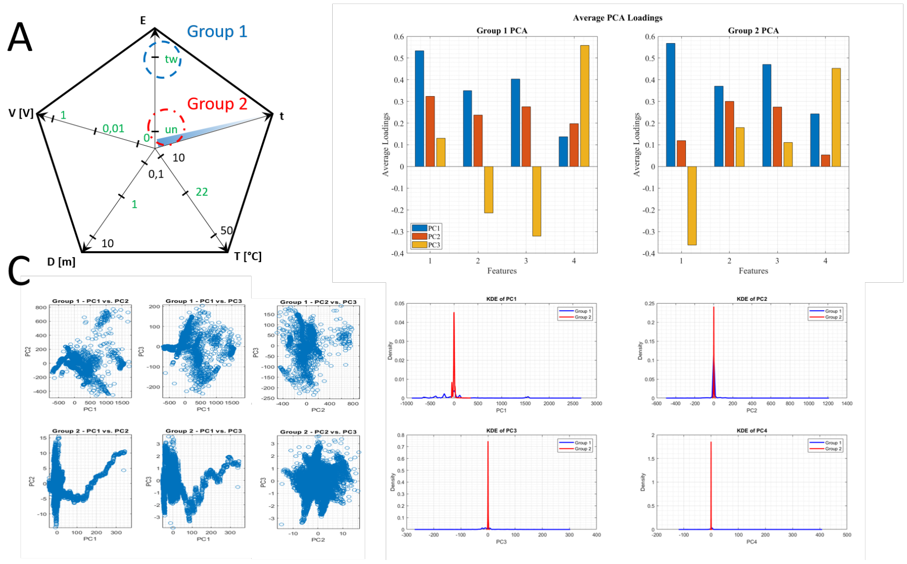

Figure 27. Machine-aided statistical analyses of the scattering matrix measurements involving the experiments performed with any stimulus, at a distance of 1 m, at 22 deg C, for the twinned versus untwinned cases. A: radargram depicting involved experiments over the entire experiment space. B: average PCA loadings of the two groups. C: aggregated PCA scores for the three principal components. D: kernel density estimates for the three principal components. $n = 96.000$.

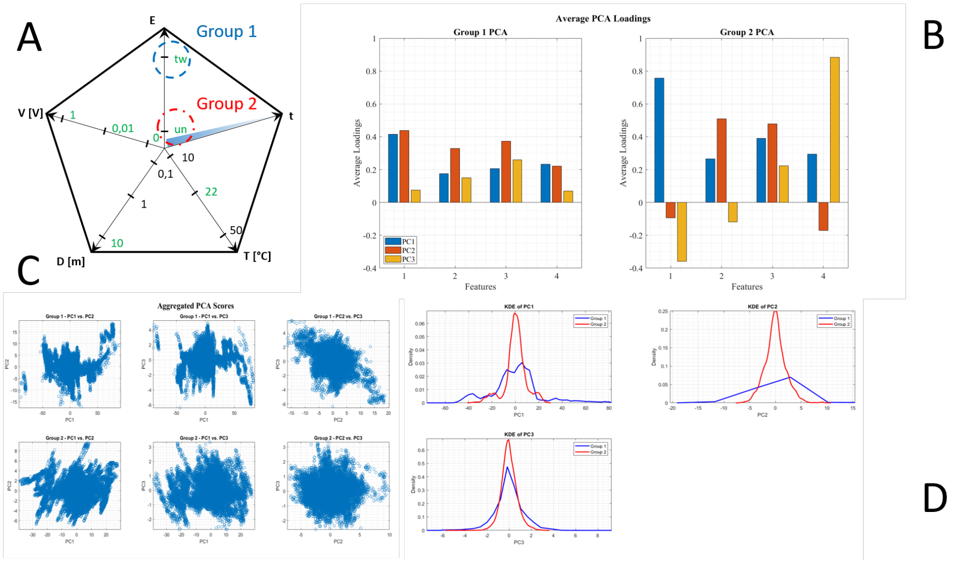

Figure 28. Machine-aided statistical analyses of the scattering matrix measurements involving the experiments performed with any stimulus, at a distance of 10 m, at 22 deg C, for the twinned versus untwinned cases. A: radargram depicting involved experiments over the entire experiment space. B: average PCA loadings of the two groups. C: aggregated PCA scores for the three principal components. D: kernel density estimates for the three principal components. $n = 96.000$.

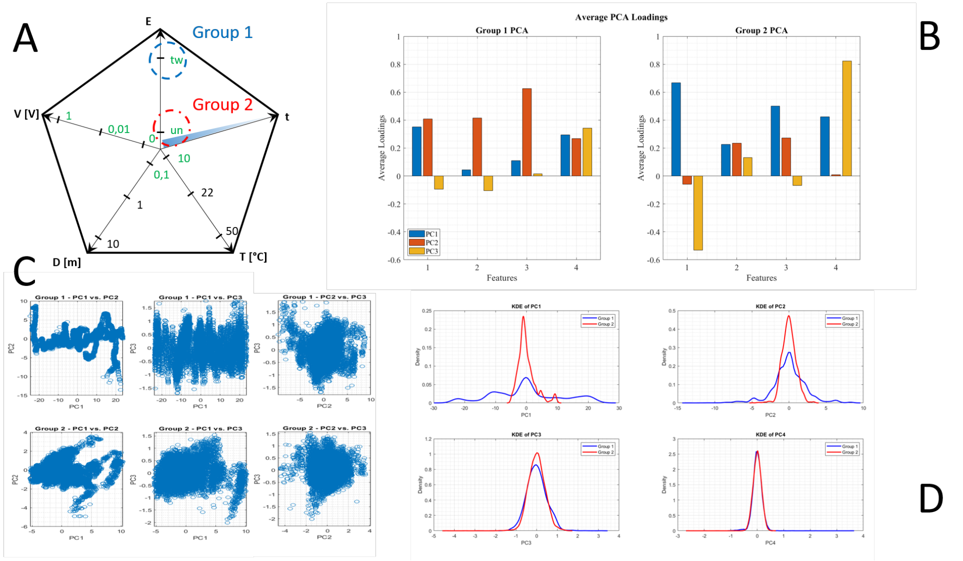

Figure 29. Machine-aided statistical analyses of the scattering matrix measurements involving the experiments performed at 10 deg C, with any stimulus, at a distance of 10 cm, for the twinned versus untwinned cases. A: radargram depicting involved experiments over the entire experiment space. B: average PCA loadings of the two groups. C: aggregated PCA scores for the three principal components. D: kernel density estimates for the three principal components. $n = 48.000$.

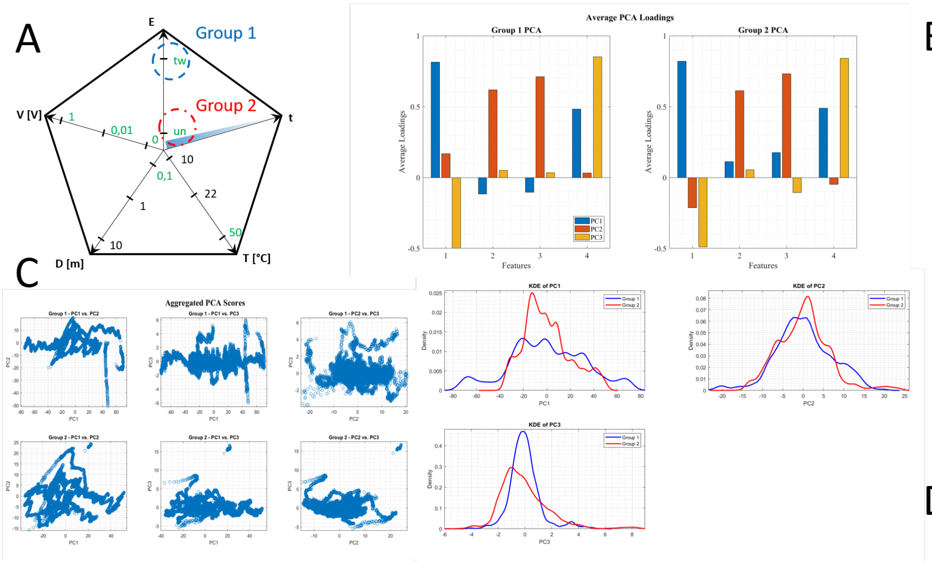

Figure 30. Machine-aided statistical analyses of the scattering matrix measurements involving the experiments performed at 50 deg C, with any stimulus, at a distance of 10 cm, for the twinned versus untwinned cases. A: radargram depicting involved experiments over the entire experiment space. B: average PCA loadings of the two groups. C: aggregated PCA scores for the three principal components. D: kernel density estimates for the three principal components. $n = 48.000$.

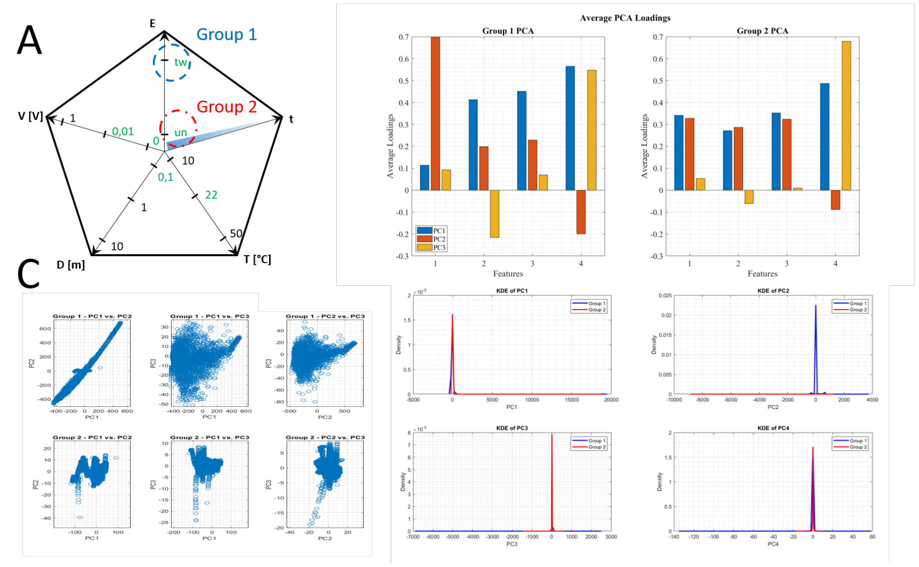

Figure 31. Machine-aided statistical analyses of the scattering matrix measurements involving the experiments performed at 22 deg C, with a 0 V or 10 mV stimulus, at a distance of 10 cm, for the twinned versus untwinned cases. A: radargram depicting involved experiments over the entire experiment space. B: average PCA loadings of the two groups. C: aggregated PCA scores for the three principal components. D: kernel density estimates for the three principal components. $n = 96.000$.

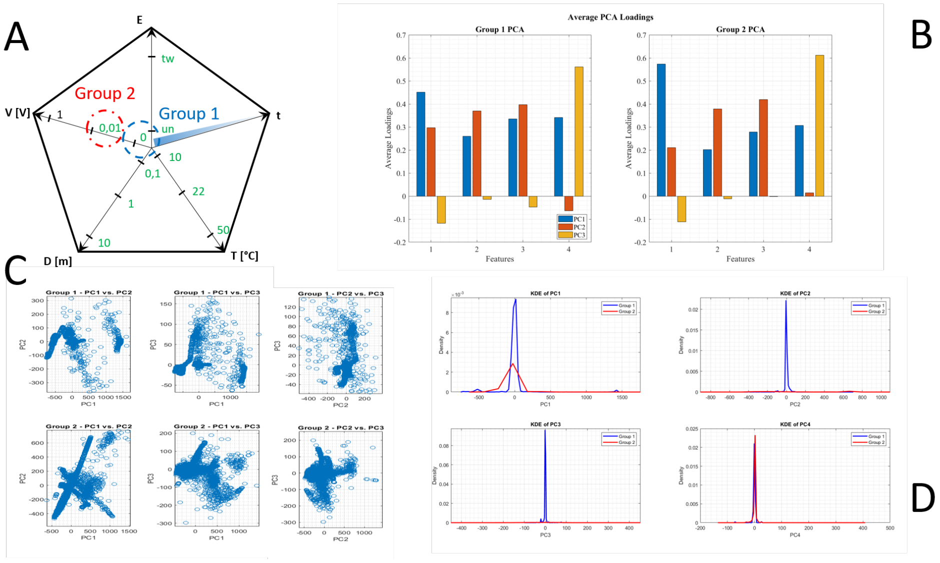

Figure 32. Machine-aided statistical analyses of the scattering matrix measurements involving the experiments performed at any twinning state, any temperature and any distance, for the 0 V versus 10 mV cases. A: radargram depicting involved experiments over the entire experiment space. B: average PCA loadings of the two groups. C: aggregated PCA scores for the three principal components. D: kernel density estimates for the three principal components. $n = 288.000$.

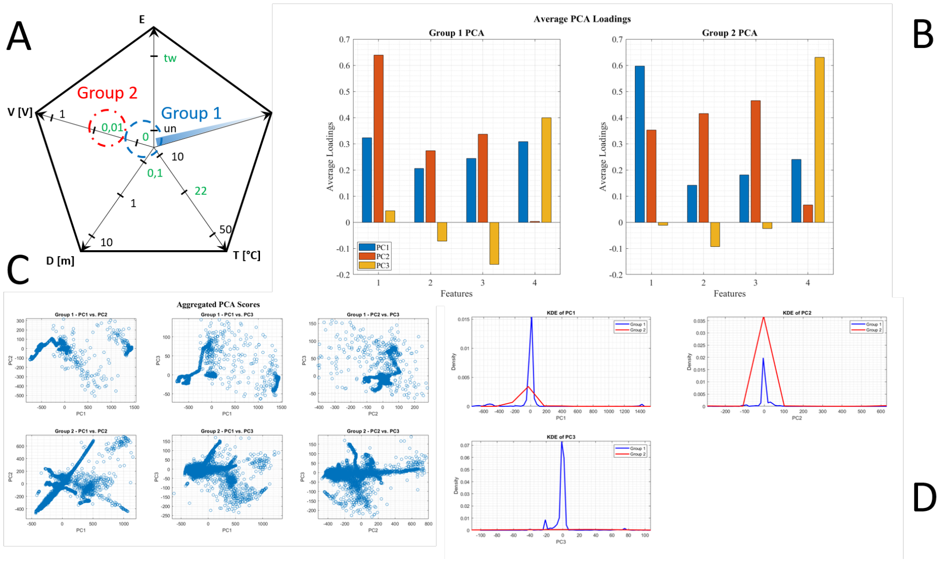

Figure 33. Machine-aided statistical analyses of the scattering matrix measurements involving the experiments performed in the twinning state, at any temperature and any distance, for the 0 V versus 10 mV cases. A: radargram depicting involved experiments over the entire experiment space. B: average PCA loadings of the two groups. C: aggregated PCA scores for the three principal components. D: kernel density estimates for the three principal components. $n = 144.000$.

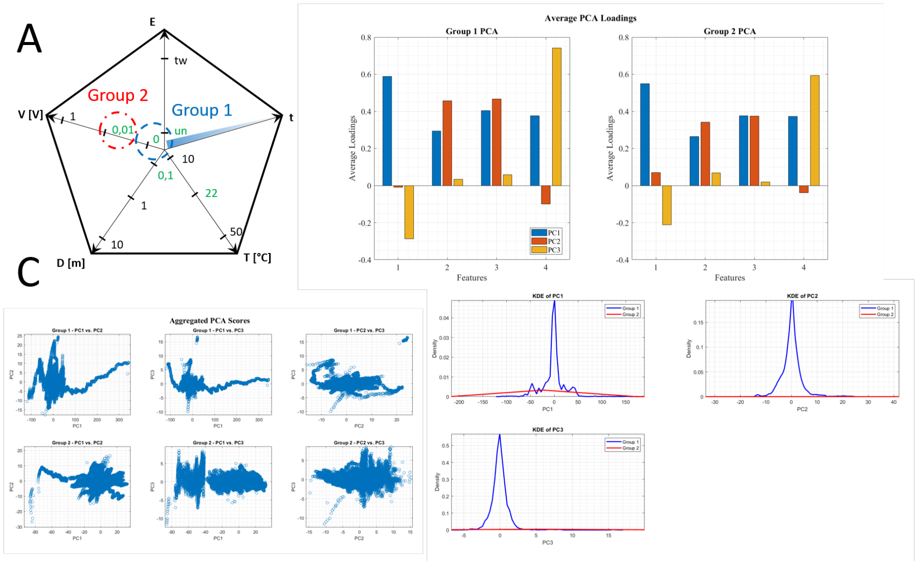

Figure 34. Machine-aided statistical analyses of the scattering matrix measurements involving the experiments performed in the untwinned state, at any temperature and any distance, for the 0 V versus 10 mV cases. A: radargram depicting involved experiments over the entire experiment space. B: average PCA loadings of the two groups. C: aggregated PCA scores for the three principal components. D: kernel density estimates for the three principal components. $n = 144.000$.

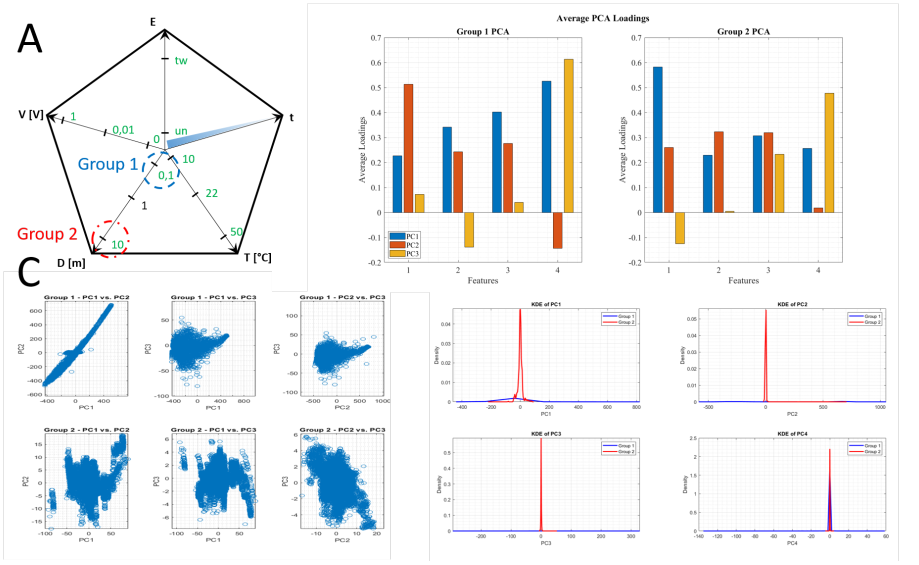

Figure 35. Machine-aided statistical analyses of the scattering matrix measurements involving the experiments performed with any twinning state, any voltage stimulus and at any temperature, for the 10 cm versus 10 m cases. A: radargram depicting involved experiments over the entire experiment space. B: average PCA loadings of the two groups. C: aggregated PCA scores for the three principal components. D: kernel density estimates for the three principal components. $n = 192.000$.

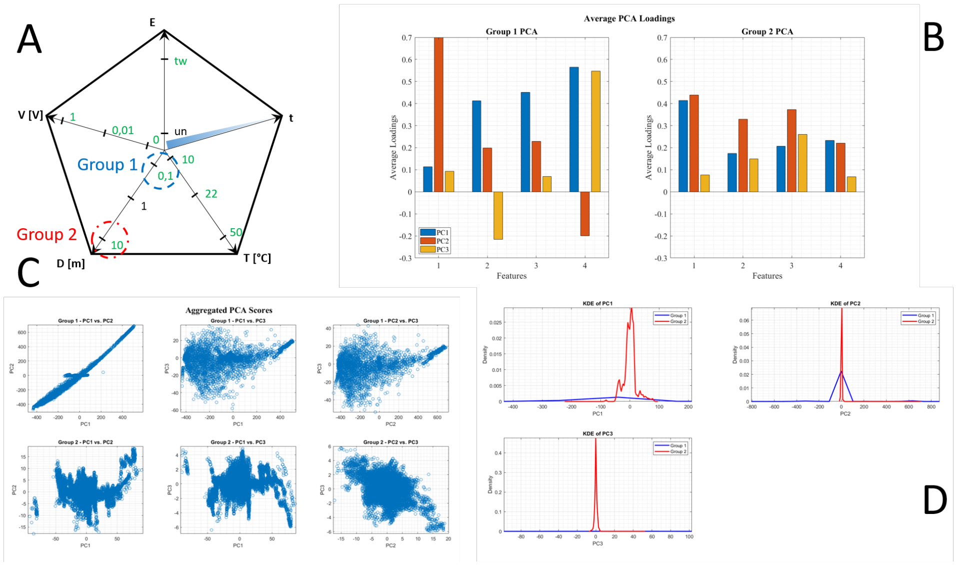

Figure 36. Machine-aided statistical analyses of the scattering matrix measurements involving the experiments performed with the twinned state, any voltage stimulus and at any temperature, for the 10 cm versus 10 m cases. A: radargram depicting involved experiments over the entire experiment space. B: average PCA loadings of the two groups. C: aggregated PCA scores for the three principal components. D: kernel density estimates for the three principal components. $n = 96.000$.

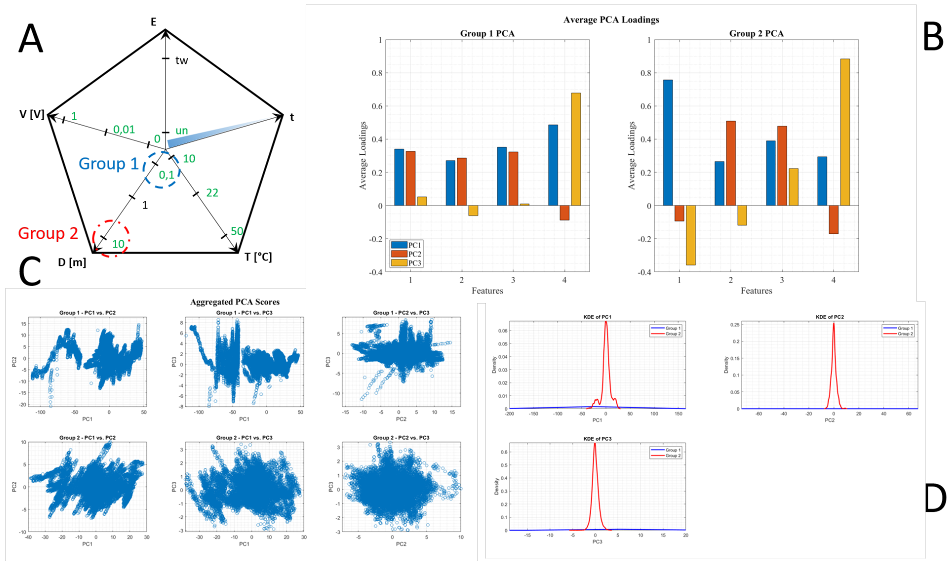

Figure 37. Machine-aided statistical analyses of the scattering matrix measurements involving the experiments performed with the untwinned state, any voltage stimulus and at any temperature, for the 10 cm versus 10 m cases. A: radargram depicting involved experiments over the entire experiment space. B: average PCA loadings of the two groups. C: aggregated PCA scores for the three principal components. D: kernel density estimates for the three principal components. $n = 96.000$.

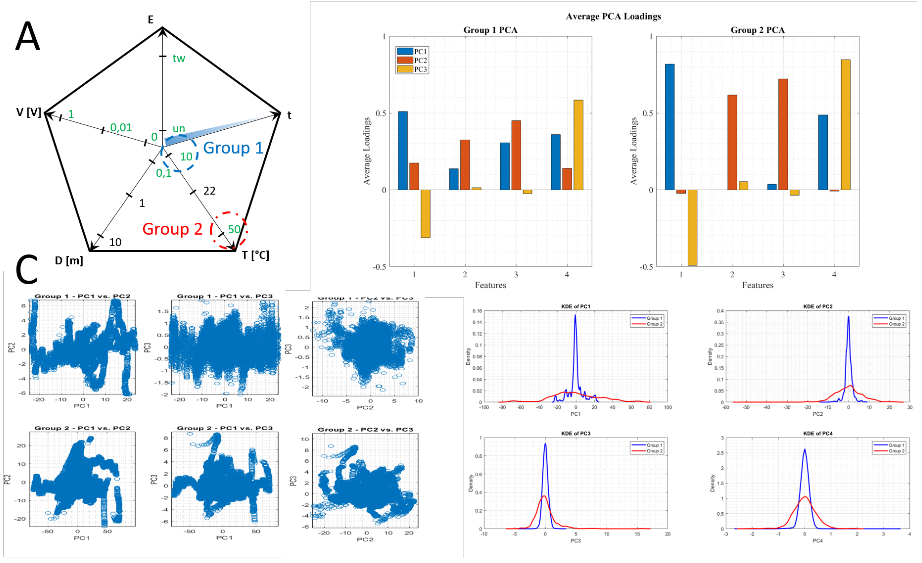

Figure 38. Machine-aided statistical analyses of the scattering matrix measurements involving the experiments performed with any twinning state, any voltage stimulus and at a distance of 10 cm, for the 10 deg C versus 50 deg C cases. A: radargram depicting involved experiments over the entire experiment space. B: average PCA loadings of the two groups. C: aggregated PCA scores for the three principal components. D: kernel density estimates for the three principal components. $n = 96.000$.

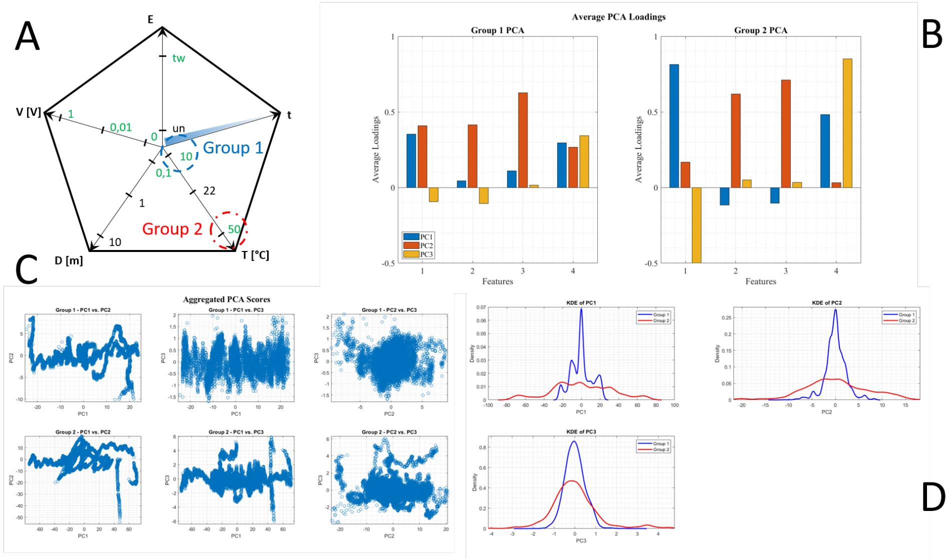

Figure 39. Machine-aided statistical analyses of the scattering matrix measurements involving the experiments performed with the twinned state, any voltage stimulus and at a distance of 10 cm, for the 10 deg C versus 50 deg C cases. A: radargram depicting involved experiments over the entire experiment space. B: average PCA loadings of the two groups. C: aggregated PCA scores for the three principal components. D: kernel density estimates for the three principal components. $n = 48.000$.

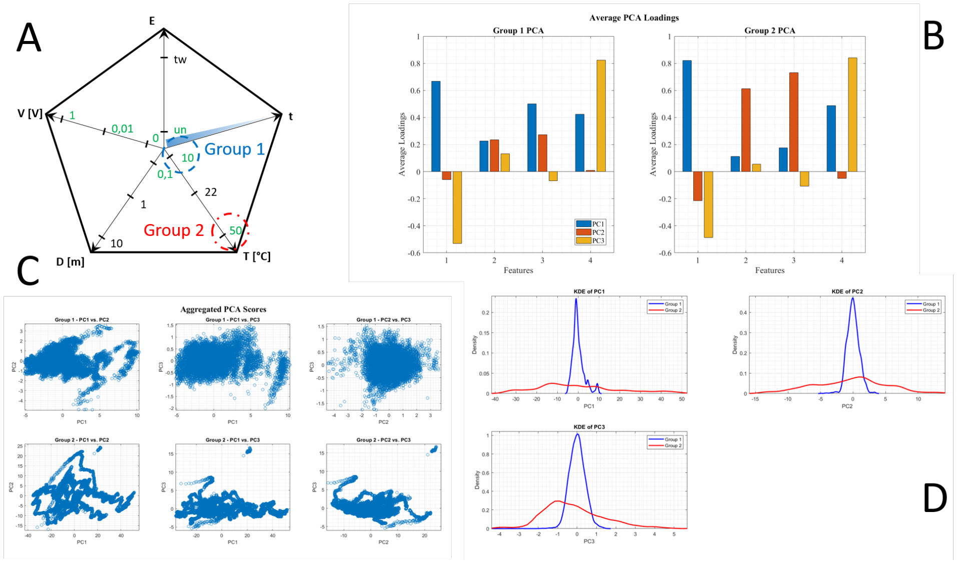

Figure 40. Machine-aided statistical analyses of the scattering matrix measurements involving the experiments performed with the untwinned state, any voltage stimulus and at a distance of 10 cm, for the 10 deg C versus 50 deg C cases. A: radargram depicting involved experiments over the entire experiment space. B: average PCA loadings of the two groups. C: aggregated PCA scores for the three principal components. D: kernel density estimates for the three principal components. $n = 48.000$.

4 Distance effect

Now, let us discuss the effects of the distance between the two vials (see Fig. 41).
The twinned case responds with a PCA featuring 2 major sign changes and 10 amplitude changes. The closest (group 1, vials kept 10 cm apart) has higher PC1 and more unbalanced features, while the farthest (group 2 , vials kept 10 m apart) has higher PC2 and more balanced features. The untwinned case responds with 2 major sign changes and 8 amplitude changes. The closest (group 1) has more balanced features, while the farthest (group 2) has more unbalanced features. Qualitatively, the PC structure of the reflection component $2,2$ and of the two transmission components $1,2$ and $2,1$ is preserved in the twinned case (short distance vials) and the untwinned state (regardless of the distance). Instead, the impedance of the twinned case with high-distance vials is something very unique.

5 Temperature Effect

Experiments run in laboratory conditions at 22 °C were performed with no temperature control, since the application of an electric field produces a negligible heating. When the voltage applied is 1 V (the highest value in this study) the associated current measured through the sample is in the order of 10 μA, therefore the energy injected into the system in 1 h of continuous stimulus is 36 mJ. If we consider no thermal loss, and a simplified situation where the vial hosts 4 mL of pure water, that energy allows us to increase its temperature by 2.2 mK ($\Delta T = Q/mc_p$ where ΔT is the temperature variation, Q is the heat, m is the liquid mass, and c_p is the heat capacity at constant pressure). The ferrofluid has both a higher specific heat and a higher density in comparison to water, therefore to a good approximation sample heating is negligible.
To conclude this statistical analysis, let us then consider the effect of temperature (see Fig. 42). The twinned case responds with a PCA featuring 3 sign changes and 7 amplitude changes. Both the cold temperature experiment at 10°C (group 1) and the hot temperature experiment at 50°C (group 2) are such that unbalanced features occur, involving a difference in both the reflection components and in the transmission ones. In the untwinned case, we see 1 sign change and 5 amplitude changes. Both the cold temperature experiment at 10°C (group 1) and the hot temperature experiment at 50°C (group 2) are such that unbalanced features occur, involving a difference in the transmission components. In contrast, the reflection ones are only very slightly affected. Most notably, the two reflection components remain affected in the twinned case while almost unaffected in the untwinned case. We can conclude that temperature has a different effect on impedance, depending on the pre-conditioning of the sample.

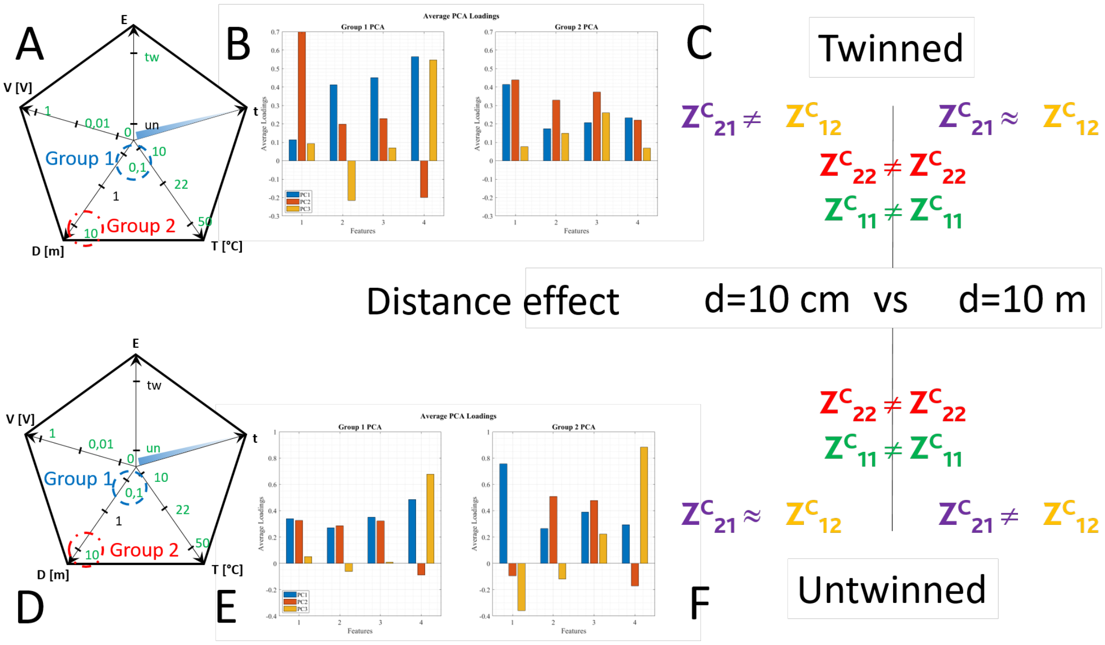

Figure 41. Effects of the distance between the two vials. Twinned case: conceptual diagram of the explored physical parameters space (A); Average PCA loadings for the two experiment groups under comparison: vials in proximity (group 1) versus vials far apart (group 2) (B); principal features summarized (C). Untwinned case: conceptual diagram of the explored physical parameters space (D); Average PCA loadings for the two experiment groups under comparison: vials in proximity (group 1) versus vials far apart (group 2) (E); principal features summarized (F). The statistical sample is composed of 192.000 entries for each of the two groups under comparison.

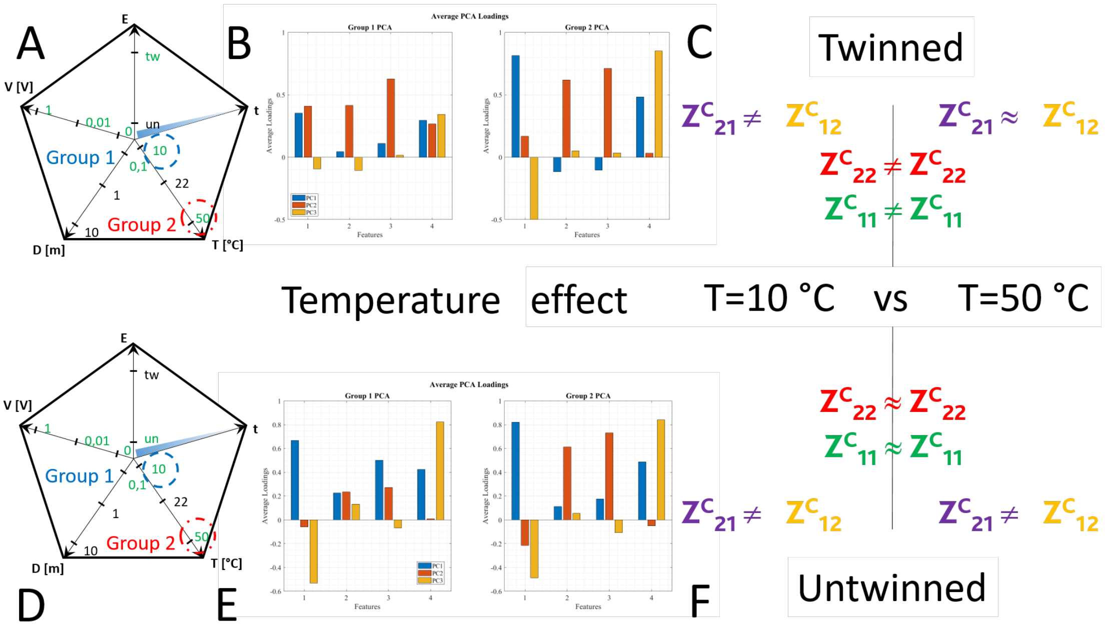

Figure 42. Effects of the temperature on the pre-conditioning state. Twinned case: conceptual diagram of the explored physical parameters space (A); Average PCA loadings for the two experiment groups under comparison: vials kept at 10 °C (group 1) versus vials kept at 50 °C (group 2) (B); principal features summarized (C). Untwinned case: conceptual diagram of the explored physical parameters space (D); Average PCA loadings for the two experiment groups under comparison: kept at 10 °C (group 1) versus vials kept at 50 °C (E); principal features summarized (F). The statistical sample comprises 96.000 entries for each of the two groups under comparison.

6 Time Effect

The time-interval effectively taken (and necessary) to perform the experiments ranges over six months. Therefore, one might compare the first set of measurements (Exp1, Exp2, and so on), regardless of other physical parameters, with the last set (Exp 6, Exp 7 with temperature variation). The outcomes of the analysis, however, would be biased by other varying conditions. To avoid such biasing conditions, both the twinned and untwinned experiments set have been performed in parallel, therefore the aging of the sample is homogeneous and linear and can be disregarded.

7 Volume Effect

Testing uneven volume distributions in the two FF reservoirs has been done, with a set of two experiments: in Exp8, vial A was filled with $2/3$ volume and connected to quasi-DC stimulus, and vial $\tilde{A}$ was filled with $1/3$ volume and connected to the VNA. In Exp9, vial A was filled with $1/3$ volume and connected to quasi-DC stimulus, and vial $\tilde{A}$ was filled with $2/3$ volume and connected to the VNA, as sketched in Figure 43. In more detail, in a first experiment we proceeded with the twinning on $V_{Tot} = 4mL$, subsequently separated the FF into $A = 8/3\ mL$ and $\tilde{A} = 4/3\ mL$, submitted vial A to the quasi-DC stimulus and vial $\tilde{A}$ to the impedance measurements according to the following conditions: 1 m distance, 0 V stimulus, and then 10 mV stimulus. The experiment was repeated without twinning preconditioning, and the results are shown in Figure 44A and B, considering for the sake of simplicity only the reflection component $Z^C_{2,2}$. We can see that in the untwinned case, the curves overlap almost perfectly (there is no correlation and hence no effect induced by voltage). In contrast, in the twinned case, the curve corresponding to 10 mV appears to shift to lower PSD in the low-frequency range when compared to the curve corresponding to 0 V, as already observed (there is a correlation and a voltage-induced effect). Worth saying that the twinning procedure results in an overall reduction of the PSD at any frequency. A similar experiment was performed by submitting to the quasi-DC stimulus vial $\tilde{A}$ (smaller volume) and to the impedance measurements vial A, with and without the twinning preconditioning. As shown in Figure 44C and D, the two cases have no relevant difference, as the curves with 0 V and 10 mV cross each other at several points. Nevertheless, the reduction of the PSD by more than one order of magnitude when the twinning is performed is found in the entire frequency range (there is no voltage-induced effect, but a measurable correlation is found).

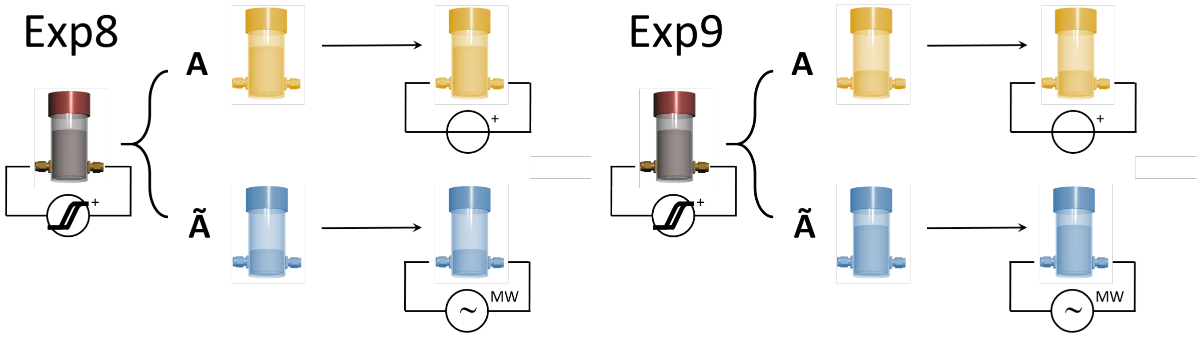

Figure 43. Schematic view of the experiments performed for the uneven distribution of liquid volumes. Reservoir A is in yellow tint, $\tilde{A}$ is in blue. The impedance measurement phase is represented by the "MW" label, the quasi-DC stimulus by the "+" label.

The following figures, from Figure 45 to Figure 48, we show the bi-logarithmic plots of the parameter Z^C_{11} for the set of two experiments of both the untwinned and twinned groups. The statistical sample shown in every graph is composed by 4.000 entries for each of the plotted curve.

8 Solvent Effect

The last set of experiments involves the use of a hydrocarbon-based ferrofluid (EFH3), Figure 44E and F. In the untwinned preconditioning, we again notice overlapping curves, while in the twinned preconditioning, there is a gap in the frequency range between 10 and 30 mHz. Comparing it to Figure 44B, where the gap opens in the range comprised between 10 and 50 mHz, we notice, therefore, a slower coupling. A typical hydrocarbon molecule found in kerosene, the dodecane, is bigger than a water molecule: a C12 linear alcane is 1.38 nm long and has a molecular weight of 170 g/mol. The distance between the two hydrogen atoms of a water molecule is 0.28 nm, and the molecular weight is 18 g/mol[9].

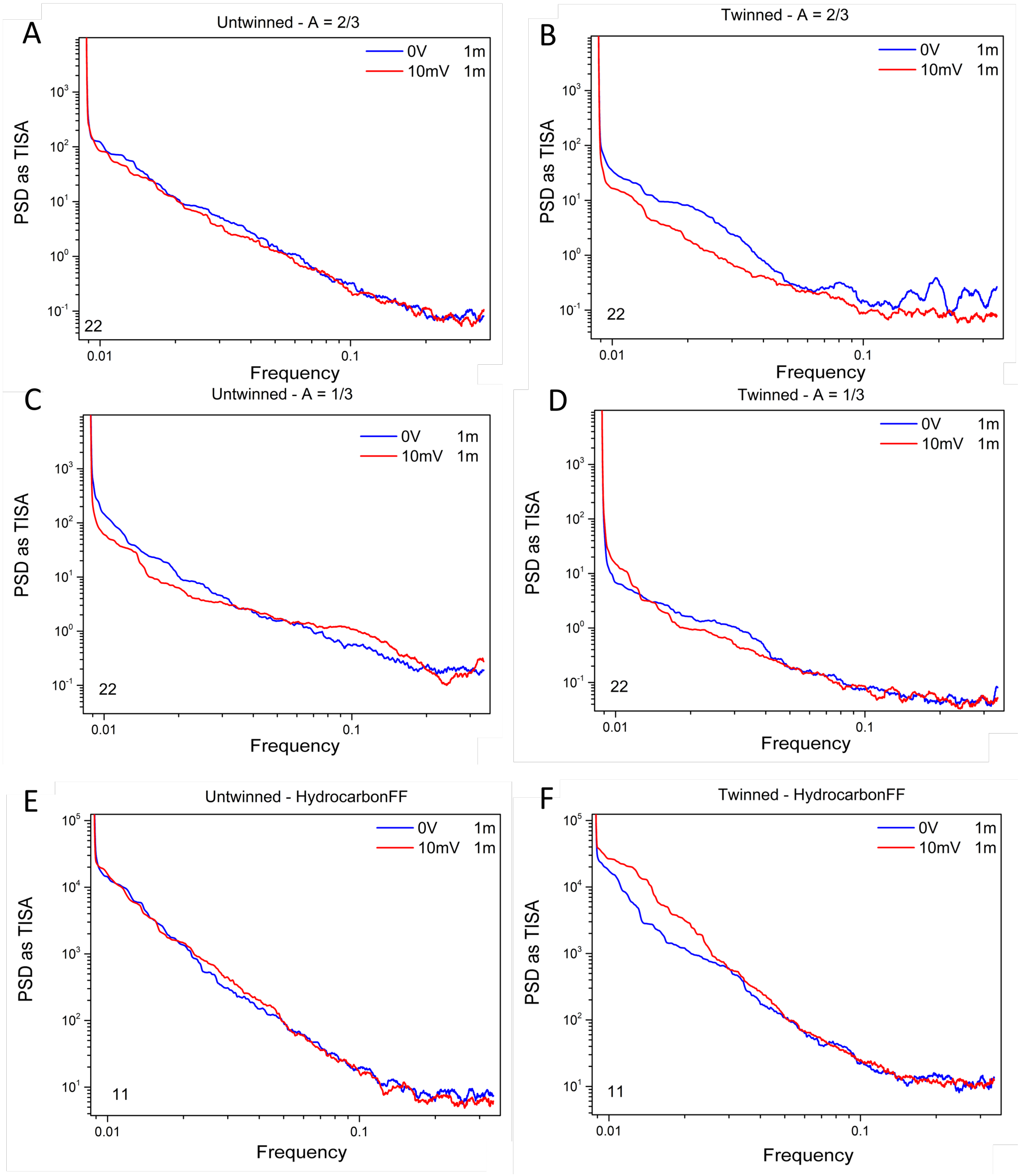

Figure 44. Effects of the uneven distribution of volumes: comparison between scattering matrix reflection parameter 2, 2 (A, B, C, D). The higher volume vial is submitted to the stimulus, untwinned (panel A) and twinned pre-conditioning (panel B). The smaller volume is submitted to the stimulus, untwinned (panel C) and twinned pre-conditioning (panel D). Effects of a hydrocarbon-based solvent: comparison between scattering matrix reflection parameter 1, 1 (E, F). The Power Spectral Density (PSD) of raw data fluctuations in the impedance "response" is plotted as TISA (Time-Integral Squared Amplitude) power, corresponding to the integral under the curve defined by the square of the raw data against time. The entire sample of 4.000 points is shown for each curve; an adjacent averaging smoothing over 101 points was used.

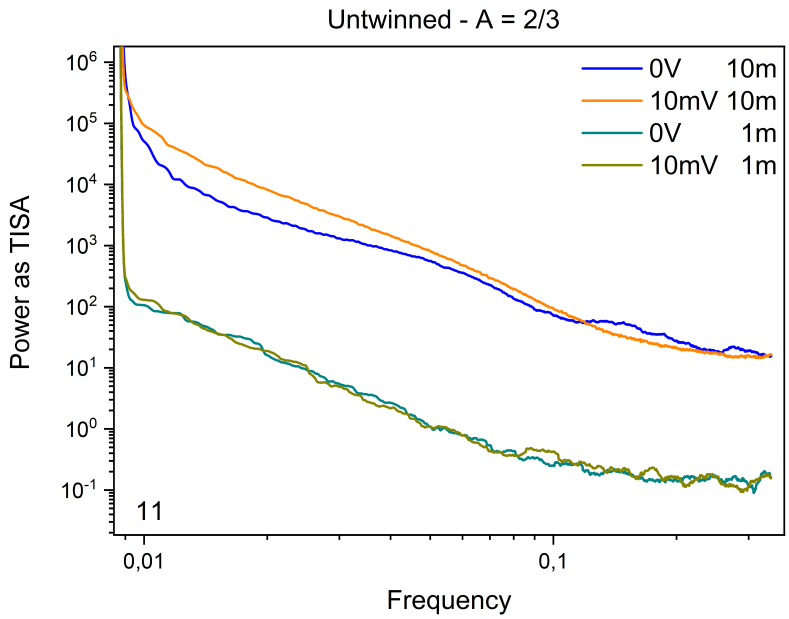

Figure 45. Z_{11}^{C} fluctuations during Exp8 in the untwinned case.

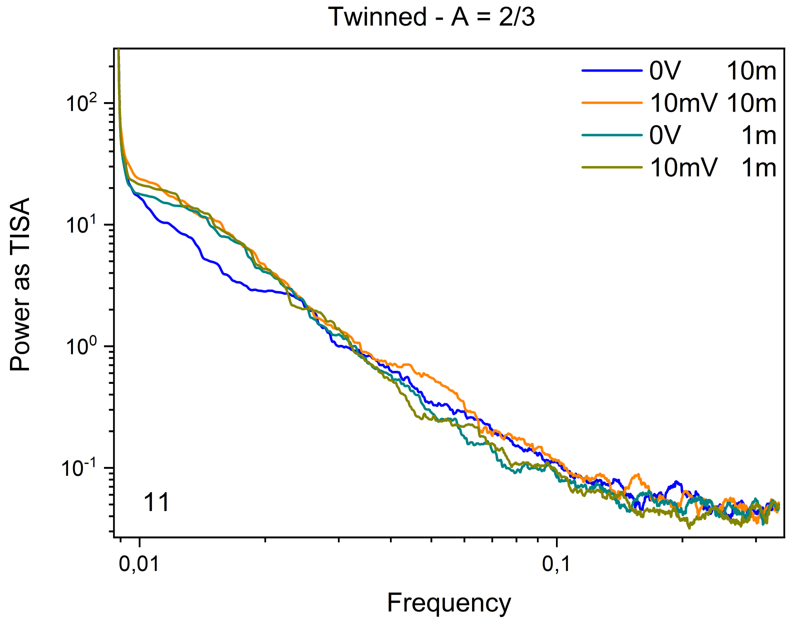

Figure 46. Z_{11}^{C} fluctuations during Exp8 in the twinned case.

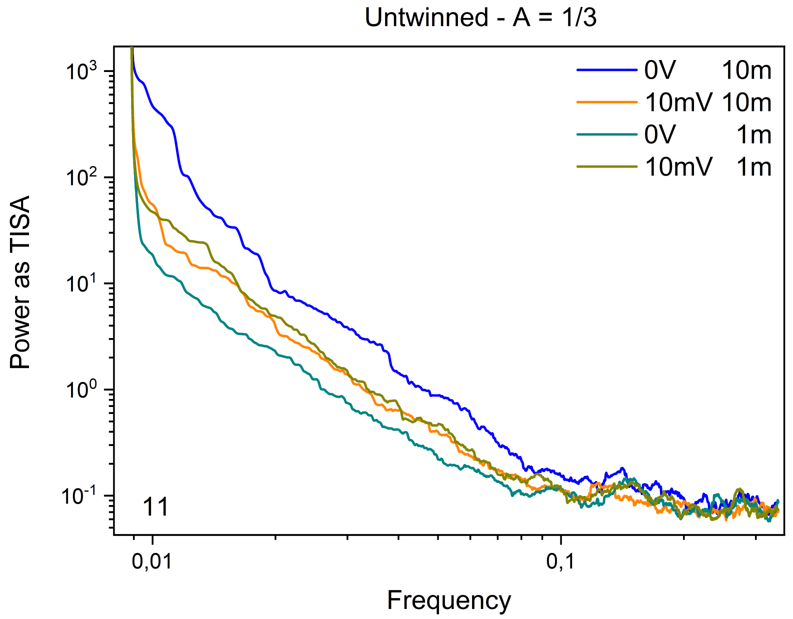

Figure 47. Z_{11}^{C} fluctuations during Exp9 in the untwinned case.

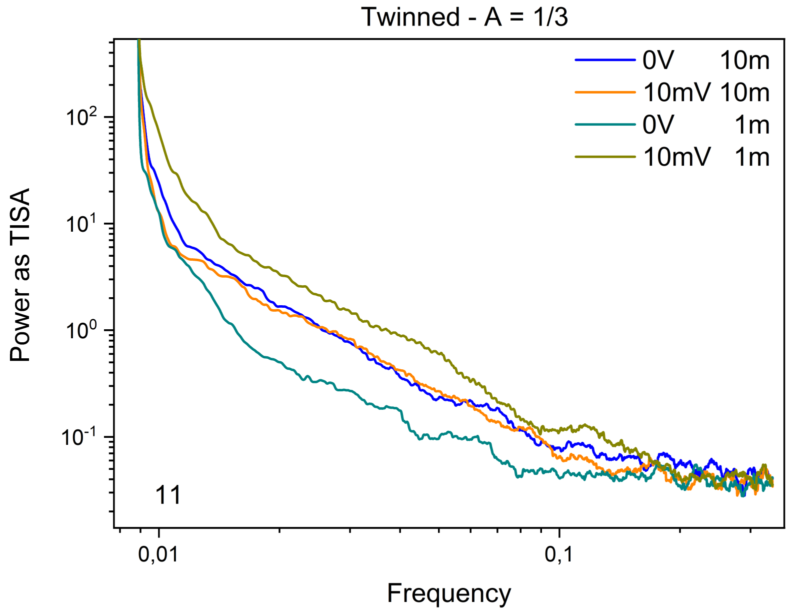

Figure 48. Z_{11}^{C} fluctuations during Exp9 in the twinned case.

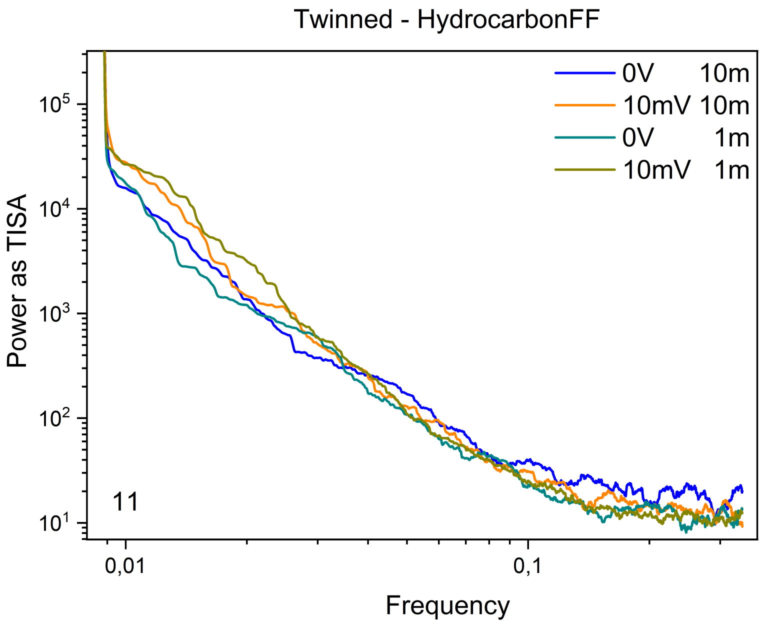

Figure 49. Z_{11}^{C} fluctuations for the twinned case, hydrocarbon-based ferrofluid.

9 Relaxation Time and Electrochemistry Effects

The electrical fluctuations set in the FF by means of the twinning pre-conditioning generate a pattern of measurable noise in the impedance, which has been monitored during long-time measurements. By following the spectral amplitude, it is possible to infer the relaxation time, as shown in Fig. 50. We have used a simple exponential decay model, which shows that the relaxation time is equal to 393.761 seconds, equal to 109h 22' 41", approximately 4d and a half. Similar measures, over a shorter timespan, have been collected on idle ferrofluid, never exposed to any electrical stimulus. To track the spontaneous fluctuations, the mean and the median of the reflection parameter Z_{11}^{C} are plotted in Fig. 50, showing that the fluctuation's amplitude is higher, and also that the impedance absolute values are quite smaller, in comparison to the state achieved after ferrofluid preconditioning.

The presence of transient electrochemical effects was checked by measuring repeatedly IV curves in the range over $\pm$ 10 Volts. Fig. 51 shows 99 cycles performed at the scanning speed of 10 V/min, where the step between each point collected is 50 mV. Electrochemical transient effects such as redox-like reactions are shown to produce characteristic features such as peaks, plateaus, and asymmetries, yet in the range relevant for our study, $\pm$ 1 Volt, only linear segments can be found. We can therefore confirm that within the voltage range explored in this study, electrochemical phenomena do not play any major role.

References

1. Singh, P. *et al.* Advances in relaxation and memory effects of magnetic nanoparticles for biomedical applications. *Prog. Mater. Sci.* **155**, 101521, DOI: https://doi.org/10.1016/j.pmatsci.2025.101521 (2026).
2. Umezawa, H., Matsumoto, M. & Tachiki, M. *Thermo Field Dynamics and condensed states* (North-Holland, Amsterdam, The Netherlands, 1982).
3. Blasone, M., Jizba, P. & Vitiello, G. *Quantum Field Theory and its macroscopic manifestations: Boson condensation, ordered patterns, and topological defects* (Imperial College Press, London, UK, 2011).
4. Goldstone, J., Salam, A. & Weinberg, S. Broken symmetries. *Phys. Rev.* **127**, 965 (1962).

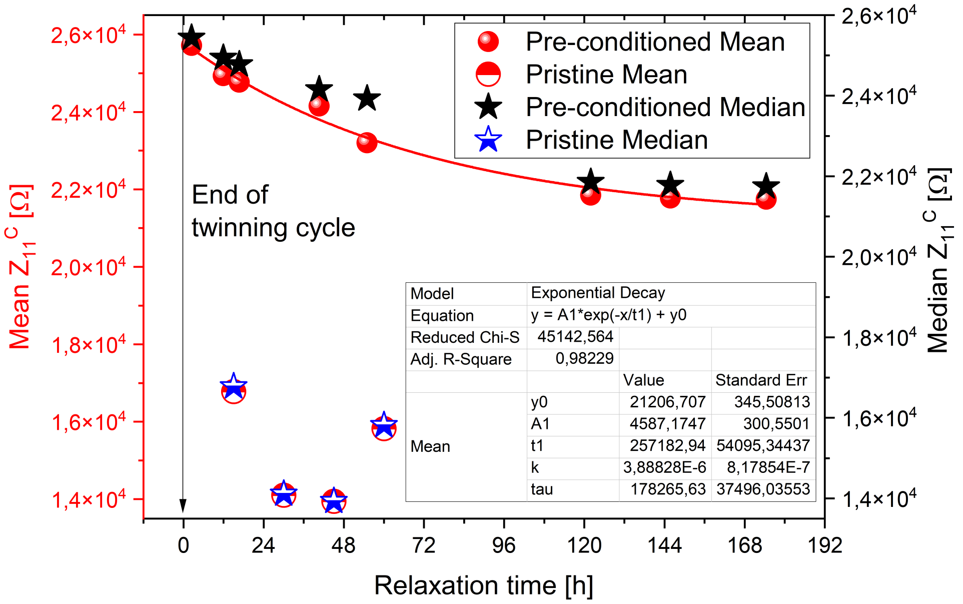

Figure 50. Left: relaxation of the impedance fluctuations measured on the scattering matrix reflection parameter $Z_{1,1}$ right after performing the twinning pre-conditioning, and in an idle state without any previous electrical stimulus given. The raw data fluctuations in the impedance are plotted as mean and median values in Ω. The fit is based on an exponential decay (red curve) and the black dashed line is a guide for the reader. Right: maximum fluctuation Power Spectral Density (PSD) as TISA (Time-Integral Squared Amplitude) power, corresponding to the integral under the curve defined by the square of the raw data against time; the red dashed lines are guides for the reader and do not correspond to a numerical fit. The entire sample is based on 1.6M experimental points.

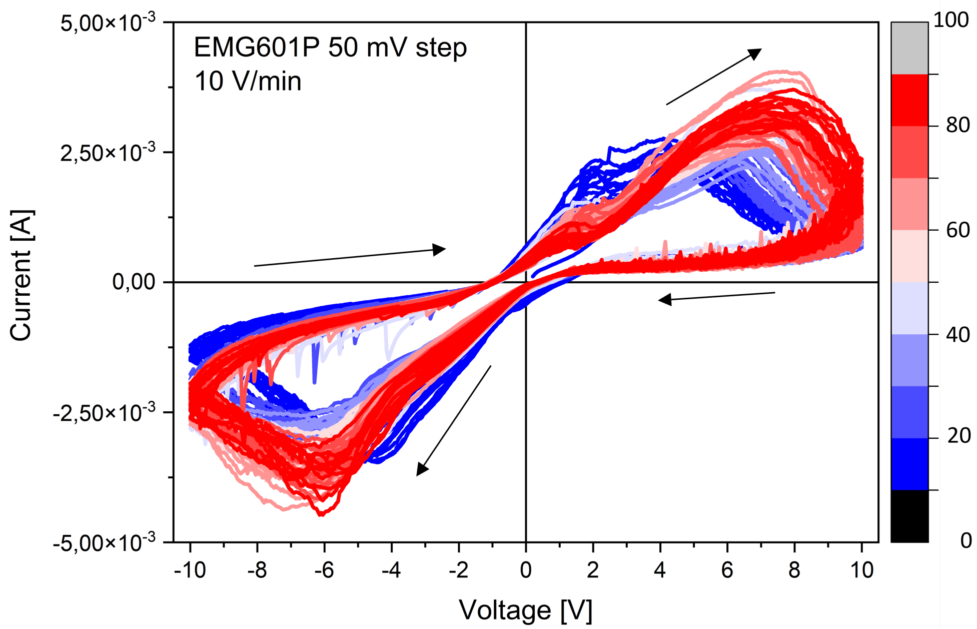

Figure 51. IV cycles collected in the range of ± 10 Volts. Fig. 51 shows 99 cycles performed at the scanning speed of 10 V/min, where the step between each point collected is 50 mV. The color code stands for the numerical ordering of the cycles between 1 and 99.

5. Itzykson, C. & Zuber, J.-B. *Quantum Field Theory* (McGraw-Hill Inc., New York, 1980).

6. Sabbadini, S. A. & Vitiello, G. Entanglement and phase-mediated correlations in quantum field theory. appication to brain-mind states. *Appl. Sci.* **9(15)**, 3203 (2019).

7. Abanin, D. A., Altman, E., Bloch, I. & Serbyn, M. Colloquium: Many-body localization, thermalization, and entanglement. *Rev. Mod. Phys.* **91**, 021001, DOI: 10.1103/RevModPhys.91.021001 (2019).

8. Binder, K. & Young, A. P. Spin glasses: Experimental facts, theoretical concepts, and open questions. *Rev. Mod. Phys.* **58**, 801–976, DOI: 10.1103/RevModPhys.58.801 (1986).

9. Dagaut, P. & Cathonnet, M. The ignition, oxidation, and combustion of kerosene: A review of experimental and kinetic modeling. *Prog. Energy Combust. Sci.* **32**, 48–92, DOI: https://doi.org/10.1016/j.pecs.2005.10.003 (2006).
